# Seeing through rose-colored glasses: How optimistic expectancies guide visual attention

**DOI:** 10.1371/journal.pone.0193311

**Published:** 2018-02-21

**Authors:** Laura Kress, Mirko Bristle, Tatjana Aue

**Affiliations:** Division of Experimental Psychology and Neuropsychology, Institute of Psychology, University of Bern, Bern, Switzerland; Goldsmiths University of London, UNITED KINGDOM

## Abstract

Optimism bias and positive attention bias have important highly similar implications for mental health but have only been examined in isolation. Investigating the causal relationships between these biases can improve the understanding of their underlying cognitive mechanisms, leading to new directions in neurocognitive research and revealing important information about normal functioning as well as the development, maintenance, and treatment of psychological diseases. In the current project, we hypothesized that optimistic expectancies can exert causal influences on attention deployment. To test this causal relation, we conducted two experiments in which we manipulated optimistic and pessimistic expectancies regarding future rewards and punishments. In a subsequent visual search task, we examined participants’ attention to positive (i.e., rewarding) and negative (i.e., punishing) target stimuli, measuring their eye gaze behavior and reaction times. In both experiments, participants’ attention was guided toward reward compared with punishment when optimistic expectancies were induced. Additionally, in Experiment 2, participants’ attention was guided toward punishment compared with reward when pessimistic expectancies were induced. However, the effect of optimistic (rather than pessimistic) expectancies on attention deployment was stronger. A key characteristic of optimism bias is that people selectively update expectancies in an optimistic direction, not in a pessimistic direction, when receiving feedback. As revealed in our studies, selective attention to rewarding versus punishing evidence when people are optimistic might explain this updating asymmetry. Thus, the current data can help clarify why optimistic expectancies are difficult to overcome. Our findings elucidate the cognitive mechanisms underlying optimism and attention bias, which can yield a better understanding of their benefits for mental health.

## Introduction

Charlie Chaplin once said that “you’ll never see a rainbow, if you’re looking down”. His famous saying implies that we do not notice the good things around us with a pessimistic attitude. Is that true? Does being optimistic or pessimistic influence which parts of our environment we pay attention to? To answer this question, we focus on the interplay between two important cognitive phenomena displayed by humans: optimism bias and positive attention bias.

Research has shown that approximately 80% of humans overestimate the likelihood of positive events and underestimate the likelihood of negative events in their future—a phenomenon called optimism bias [1,2]. In contrast to healthy people, who tend to be overly optimistic, patients suffering from depression do not display an optimism bias and are rather realistic about their future [3]. Therefore, optimism bias is broadly viewed as a necessary psychological adaptation that promotes mental health [3,4] and ensures motivation for goal-directed behavior [5,6]. However, being too optimistic can also have dramatic negative consequences and might lead to criminal or addictive behavior, especially when people underestimate the negative consequences of committing a crime or consuming drugs [7,8].

Notably, optimism bias is displayed even considering contradictory information [9]. People find it more difficult to adapt their expectancies regarding important future life events when they receive feedback that is worse than expected (bad news such as that the average likelihood to incur serious health problems is higher than the individual had initially predicted for herself) than when it is better than expected (good news such as that the average likelihood to incur serious health problems is lower than the individual had initially predicted for herself) [9]. Such selective updating could explain why optimistic outlooks are maintained over time and shows that optimism-related processing in healthy individuals is distinct from other forms of future expectancies (i.e., pessimism) in terms of robustness.

Although selective updating has been proposed to maintain optimism bias, the cognitive mechanisms underlying optimism bias and its pervasiveness–even in light of existing contradictory information–are still unclear. We know that optimism bias exists but we do not know precisely why it exists and how it is maintained. Here, we suggest that investigation of the interactions between different types of cognitive biases can provide information about these mechanisms. We argue that examining optimistic expectancies in relation to attention deployment could yield a better understanding of optimism bias and its benefits in everyday life as in the clinical domain.

Our postulate that biased expectancies and attention deployment are interdependent is based on two points; the first is observational and the second is theoretical in nature. First, a positivity bias not only exists in regard to future expectancies (as in optimism bias) but also in regard to visual attention: Positive and rewarding stimuli attract people´s visual attention more than neutral (and sometimes negative) stimuli do [10,11]. This has been shown by more rapid reaction times (RTs) and captured eye movements to rewarding than neutral information in different attention paradigms. Happy faces, for instance, have been proposed to "pop out" of crowds in visual search tasks [11]. Moreover, positive attention bias, comparable to optimism bias, has been demonstrated to hold important implications for mental health [12].

Preferably attending to positive rather than neutral stimuli enables people to efficiently detect events in an environment in which several stimuli compete for access to their limited attention resources. If people´s attention is biased toward positive stimuli in their environment, they are more likely to perceive chances to maximize beneficial output. From an evolutionary point of view, this could contribute to fitness for survival [13,14]. However, how biased expectancies relate to biased attention (e.g., whether expectancies modulate biased attention or vice versa) has not been investigated. The discovery of interactions between the two biases under investigation would yield a better understanding of optimism bias and positive attention bias.

Second, according to the combined cognitive biases hypothesis, negative cognitive biases (e.g., in attention, interpretation, and self-imagery) usually interact and mutually enforce each other [15] (see [12] for considerations on the combined cognitive biases hypothesis in depression and [16] for the interplay of expectancies and attention in anxiety). This theory mainly focuses on associations between negative biases. Recently, similar interactions between different cognitive biases have been proposed in the positive domain [17]. Revealing such causal relations between cognitive biases in the positive domain allows for investigation of why positive cognitive biases exist and how they are maintained over time. These investigations broaden our knowledge about normal functioning and the development of psychological disorders as well as their treatment and uncover divergences and commonalities between cognitive bias interactions in health and psychopathology.

Investigating the relation between optimism bias and positive attention bias is especially interesting because causal influences of optimistic expectancies on attention can elucidate how certain stimuli are processed when people are optimistic (e.g., concerning their processing depth). For instance, optimism-driven attention deployment could directly explain important phenomena shown in optimism bias such as the selective updating described above [9]. This selective updating could be caused by optimistic expectancies shifting attention to rewarding (i.e., good news) rather than punishing (i.e., bad news) evidence, thereby determining the processing depth of the respective evidence. This should have retroactive, stabilizing effects on the initial optimism displayed. For example, it is conceivable that people displaying optimism bias might be particularly attentive when being told that their likelihood to incur a serious health problem is lower than they had initially predicted (good news) whereas they are less attentive when being told that their likelihood to incur a serious health problem is higher than they initially predicted (bad news). This will lead to a deeper processing of the good news (e.g., by further thinking about the new information). Consequently, people could selectively integrate good news when updating their expectancies and neglect bad news. Asymmetric attention deployment to good vs. bad news following optimistic expectancies would thus have significant feedback effects on these initial expectancies, thereby stabilizing optimistic tendencies in the long run.

If one assumes that the processing depth of rewarding or punishing stimuli can be influenced by optimistic expectancies, it is especially important to distinguish between various stages of attention deployment (e.g., initial orientation and maintenance of attention) and determine at which stage such differential processing takes place. The use of eye tracking allows for such a distinction [18] and could therefore reveal insights into the concrete attentional mechanisms that are crucial for selective updating processes in optimism bias. For instance, one could imagine that, when being optimistic, people initially orient their attention (primarily an automatic process) toward both good and bad news but later maintain attention (primarily a controlled process) selectively on good news (see [19] for differences in attention orientation and maintenance on emotional stimuli shown by dysphoric participants). Such a finding would have crucial implications for a more profound understanding of the concrete nature of biased expectancy-attention interplay in healthy individuals and may fundamentally inspire psychotherapy. For instance, it could uncover the specific mechanisms to be targeted in depressive patients, who do not show a beneficial updating asymmetry [3].

There is no substantial empirical evidence for a causal link between biased optimistic expectancies and attention. However, examples in the literature show that expectancies can guide visual spatial attention in the positive domain [20–22]. Spatial attention could be influenced by expectancy cues when using motivationally relevant (rewarding) target pictures in a covered attention shift paradigm [20]. Participants reacted faster to cued food targets when they were motivationally relevant (i.e., when participants were hungry compared with when they were full). The same effect was not present for motivationally irrelevant tool targets. Other findings suggest that attention to happy faces can be modulated in a top-down manner through instructions that presumably impact expectancies [21,22] (see [23–25] for similar effects with neutral stimuli). These findings are in line with predictive coding theory [26,27], which states that expectancies allow people to create a mental template of expected information that is then compared with sensory input. During this comparison, attention might be biased to information that fits with the created template. However, it is important to note that the respective expectancies in the abovementioned studies [20–22] were unrelated to optimism and pessimism (e.g., because participants were explicitly instructed to search for a happy or sad face [21,22]).

Even though there are no studies directly linking optimism bias and positive attention bias, a few studies examine the link between trait optimism (typically assessed with the Life Orientation Test (LOT-R [28]; or similar personality scales) and attention deployment. Whereas trait optimism describes a stable disposition of having an optimistic yet not necessarily unrealistic life orientation, optimism bias describes unrealistic expectancies regarding specific future situations that can be manifold (e.g., concerning health, relationships, and wealth). Although trait optimism and optimism bias are different constructs, trait optimism might increase an individual’s readiness to demonstrate optimism bias in specific circumscribed situations [1,6]. Notably, trait optimism has been related to an attention bias toward positive and away from negative stimuli, shown by altered reaction times in a Stroop paradigm [29,30] and biased eye movements [31,32]. Unfortunately, all reviewed results on the link between trait optimism and attention are of correlational nature and thus do not provide information on causal relationships.

To our knowledge, only one study attempted to manipulate participants’ optimistic expectancies experimentally and provide information about the direction of influence between optimism and attention. Peters and colleagues (2015) induced state optimism, measured by the Future Expectancies Scale, in half of their participants using the Best Possible Self (BPS) manipulation. During this BPS manipulation, participants imagined a future life in which everything had gone well while the other half of the participants underwent a neutral control manipulation. Next, both groups performed a passive viewing task in which their attention deployment was assessed. Although optimism manipulation did not influence gazing behavior in general, post-hoc analyses showed that, in contrast to non-responders, participants whose state optimism increased after the state optimism or control manipulation gazed significantly shorter at angry faces and nearly significantly longer at joyful faces than participants whose state optimism did not increase [32]. These data indicate that state optimism, which most likely instigates optimism bias, might bias attention deployment toward positive and away from negative stimuli. However, additional research is needed to substantiate such a causal association.

It is generally difficult to directly manipulate optimism bias because (a) it is unclear how to reliably provoke such a bias across individuals and situations because it depends on a combination of many different aspects (some of which are impossible to manipulate, e.g., personal experience, individual preferences) [2]; (b) a bias is always relative to some other measure (e.g., overly optimistic expectancies in comparison with other people or reality), which makes it difficult to be evoked and measured; and (c) some types of manipulations may rely on simultaneous control of expectancies and attention. Thus, research on optimism bias and other cognitive biases has mostly been of a correlational nature. A first step toward demonstrating that optimism bias and positive attention bias are causally associated may be to demonstrate that optimistic expectancies (which are not necessarily biased) influence attention deployment and/or vice versa [32].

In the current studies, therefore, we manipulated optimistic and pessimistic expectancies that are present in optimism bias [33] (instead of operationalizing optimism bias per se) and investigated their respective causal influences on attention deployment. If the findings show that variations in experimentally induced expectancies successfully generate changes in visual attention, it may be assumed that biases in expectancies can generate biases in attention. In our studies, expectancies were manipulated by verbal cues presented prior to a visual search task (see [23,25] for studies using a similar paradigm with neutral and threatening stimuli). During the presentation of expectancy cues, the change in participants’ pupil diameter (measure of autonomic arousal [34]) was measured to demonstrate that cues elicited an affective response that can be attributed to optimistic and pessimistic expectancies. During the visual search task, two different components of attention were measured. First, attention orientation was measured by (a) RTs in the visual search task and (b) time to first hit on a target revealed by eye tracking data (i.e., the moment when the participant’s gaze was registered to be first on the target). These measures of attention orientation were intended to investigate more automatic effects of optimistic expectancies on attention deployment. Second, attention maintenance during the visual search task was measured by how long participants looked at a target half a second after the first hit. Attention maintenance reveals information on how deeply stimuli signaling reward and punishment were processed following optimistic expectancies [35,36] and can provide information on more controlled attention processes that explain the selective updating shown in optimism bias. We chose to acquire eye tracking in addition to RTs as it represents a more direct measure of attention and can reveal effects that are not visible in RT data [37]. Moreover, we measured participants’ self-reported comparative optimism bias [2] to determine how individual differences in self-reported optimism bias are related to optimism-induced attentional biases revealed by our experiments.

We conducted two experiments using different stimuli (Experiment 1: happy, sad, and neutral faces and Experiment 2: letters of different colors) in the respective visual search tasks. The letter experiment was conducted in addition to the first experiment because the happy and sad faces themselves contain fixed valences. In the second experiment, valence was assigned by verbal instructions to neutral letter stimuli and "reward" and "punishment" connotations for the different stimuli were balanced across participants to avoid rigid stimulus-valence associations.

The aim of the present studies was to determine if experimentally induced optimistic and pessimistic expectancies regarding future gains and losses causally impact attention deployment to stimuli signaling reward (i.e., gain) and punishment (i.e., loss). For both experiments, we hypothesized that (1) gain and loss cues presented during the expectancy phase of the experiments elicit an affective response that can be attributed to optimism and pessimism (manipulation check). We hypothesized a larger increase in pupil diameter when participants were presented with gain or loss cues than when they were presented with ambiguous cues (control condition that should not contain a specific affective dimension). This hypothesis was drawn from past research that has shown differential pupil diameter change for gain and loss cues compared with neutral cues [38].

Furthermore, we hypothesized that (2) induced optimistic expectancies guide attention toward reward compared with punishment whereas pessimistic expectancies guide attention toward punishment compared with reward (differences between attention orientation and maintenance were examined exploratively as we did not have specific hypotheses). We anticipated that (2a) gain cues enhance attention to gain targets in comparison with loss cues, (2b) loss cues enhance attention to loss targets in comparison with gain cues (cue congruency hypothesis), (2c) gain cues enhance attention to gain in comparison with loss targets, and (2d) loss cues enhance attention to loss in comparison with gain targets (target congruency hypothesis).

Moreover, we hypothesized that (3) optimistic expectancies guide attention more toward reward compared with punishment than pessimistic expectancies guide attention toward punishment compared with reward because optimistic expectancies have been shown to be more robust (i.e., more resistant against disconfirming feedback) than pessimistic expectancies [9] (optimism robustness hypothesis). Therefore, even though we hypothesized an influence of pessimistic expectancies on attention toward punishment compared with reward, we anticipated this influence to be much weaker than the influence of optimistic expectancies on attention to reward compared with punishment.

Last, we hypothesized that (4) this optimism robustness in attention (i.e., stronger guidance of attention to reward compared with punishment through optimistic expectancies than to punishment compared with reward through pessimistic expectancies) is positively related to participants’ self-reported comparative optimism bias (comparative optimism bias hypothesis) [2].

## Experiment 1: Methods and materials

### Participants

Thirty-two healthy psychology students recruited via the participant pool at the University of Bern took part in this RT and eye tracking study. Wearing hard contact lenses or reporting the use of psychoactive substances served as exclusion criteria. Participants had normal or corrected-to-normal vision and were reimbursed with course credit and 5 Swiss francs for participation. One participant was excluded because of a technical error in data logging, leaving a final sample of 31 students (4 male, age: *M* = 21.19 years; *SD* = 1.60 years; range = 19 – 26 years). All participants gave written informed consent according to the ethical standards guidelines of the Declaration of Helsinki and were told that they could end the experiment at any time. All procedures were approved by the local ethical review board of the Faculty of Human Sciences at the University of Bern, Switzerland.

### Stimuli

**Fig 1:**
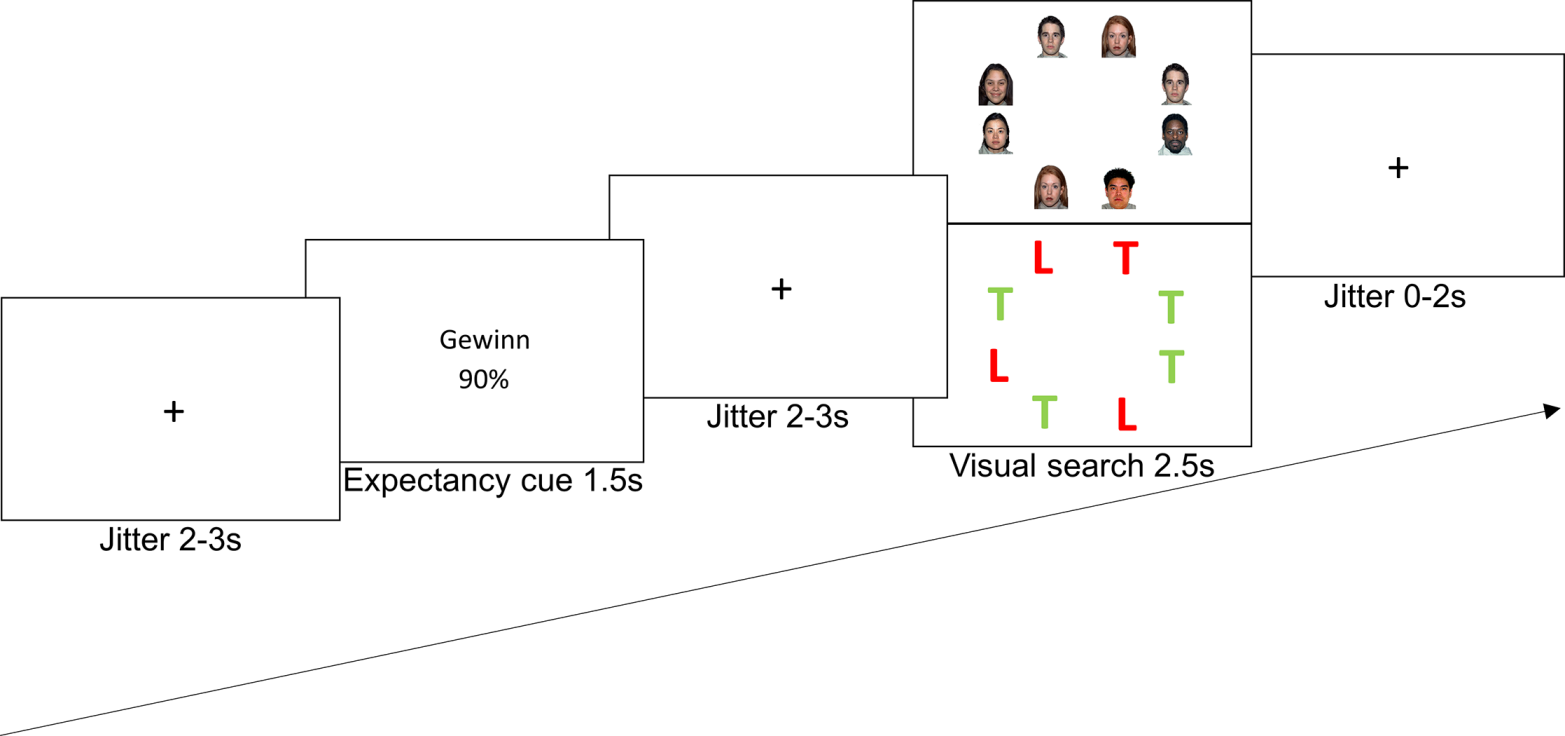
Task sequence. An example of a gain-90% cue (Gewinn [German word for gain] 90%) followed by a search array depicting a gain target (happy face [Experiment 1, top] or red T [Experiment 2, bottom]). Participants were told that the cues described the likelihood of seeing a gain or loss target in the search array. They were also told that they would gain (lose) 25 Swiss cents in addition to a starting amount of 5 Swiss francs when seeing a gain (loss) target. Participants were asked to respond as quickly and accurately as possible according to the target (i.e., gain or loss target). Due to the copyright regulations of the NimStim face stimuli [39], faces that were not used in Experiment 1 were displayed for illustration (top) and two faces are shown twice although the same face was never repeated in any trial of Experiment 1.

Visual search task (attention): Forty-eight face stimuli taken from the NimStim Face Stimulus Set [39] served as stimuli. Sixteen different faces (half male and female) each displayed happy, sad, and neutral facial expressions. In every trial, eight faces were shown on a white background on a circle around the position where the fixation cross had been presented before (Fig 1, top). The participants’ task was to find the deviant (happy or sad) target face among seven neutral distractor faces. Happy and sad faces appeared equally probable in any of the eight different locations on the circle and signaled gain (i.e., reward) and loss (i.e., punishment) of money, respectively. The stimuli were matched for luminance and contrast and displayed in color.

Cues (expectancy): Three different verbal cues were presented: “gain 90%”, “loss 90%”, or “gain loss 50%” (“loss gain 50%” for half of the participants). These cues indicated the probability that the to-be detected target in a subsequently presented search array is a happy or sad face. The gain 90% (loss 90%) cue condition referred to a probability of 67% (64 trials) that there would be a happy face (sad face) among seven neutral faces in the subsequent search array. In the remaining cases, a sad face (happy face) was presented (32 trials). In the 50% cue condition, happy and sad faces were equally likely to be the target in the search array (64 trials, 32 happy face targets and 32 sad face targets). This 50% cue was included as a control condition inducing ambiguous expectancies with maximum uncertainty. E-Prime 2.0 Professional (Psychology Software Tools, Pittsburgh, PA) was used to present stimuli and record the participants’ responses.

### Experimental procedure

After providing written informed consent, participants read the instructions in which the experiment was described as a gamble task with the opportunity to gain or lose money. They were told that they would gain 25 Swiss cents in addition to a starting amount of 5 Swiss francs upon seeing a happy face in the visual search array and lose 25 Swiss cents upon seeing a sad face. Participants were told that the cues in the beginning of each trial described an average probability of a happy or sad face being presented subsequently but the computer randomly picked a target out of a pool of 100 targets (for 90% gain [loss] cues, this pool consisted of 90 happy [sad] and 10 sad [happy] faces). Therefore, the real probabilities could differ from the average value displayed as the expectancy cue. Before starting the experiment, participants performed six practice trials to become familiar with the task.

Fig 1 (top) shows the timing and sequence of one example trial. In each trial, participants were presented a fixation cross for 2000–3000 ms followed by a cue word that was presented for 1500 ms. The cue indicated how probable it was that the to-be detected target in the subsequently presented search array would be a happy or a sad face (see the preceding section for details regarding the expectancy cues). After the cue was presented, another fixation cross appeared for 2000–3000 ms. The search array consisting of eight pictures (seven neutral faces and either a happy [gain] or sad [loss] face) was then shown for 2500 ms. During the visual search task, participants had to indicate whether the target was presented on the left or right side of the screen by pressing 1 or 2 on the number pad of the computer keyboard. The participants were instructed to react as quickly and correctly as possible. After the detection period had elapsed, another fixation cross was presented for 0–2000 ms before the next trial.

Two hundred forty-four experimental trials were presented in random order in four blocks of 61 trials with short pauses in between. The frequencies of trials of different types (cues, targets) were comparable between blocks. In total, participants both gained and lost 32 Swiss Francs, leaving them with the starting amount of 5 Swiss Francs. Participants were not informed about the progression of their gains and losses during the experiment.

After the experiment, participants completed a post-experimental questionnaire consisting of specific questions about how they perceived and conducted the task (e.g., whether they had employed a specific strategy during the search task [and if so, which strategy], see S1 Analysis for further details). Participants also completed different personality questionnaires (LOT-R [28], Comparative Optimism Scale [COS; 2], Future Expectancy Scale [40], Satisfaction With Life Scale [41], Positive And Negative Affect Schedule [42], Emotion Regulation Questionnaire [43], Behavioral Inhibition System/Behavioral Activation System Scales [44], 10-Item Big Five Inventory [45]), were debriefed, and received their “gain” of 5 Swiss francs.

### Eye tracking

Eye tracking data were acquired with a Tobii Pro X2-60 remote eye tracker (Tobii AB, Stockholm, Sweden). The system used the corneal reflection light source (corneal reflex method) to measure the eye’s orientation. Eye movements were recorded binocularly with a 60 Hz sampling rate and an accuracy of .4°. The system was controlled by Tobii Studio (version 3.1.6) to register ocular movements.

### Manipulation check

As a manipulation check, affective arousal during the presentation of expectancy cues was measured by the change in participants’ pupil diameter. For pupil diameter analysis, five 0.5-s intervals from 0 to 2.5 s after cue onset were considered. Pupil diameter during the 0.5 s before the appearance of the cue (presentation of fixation cross) served as baseline. Pupil diameter baseline scores were subtracted from the scores during cue presentation to obtain difference scores describing changes from the presentation of the different cues. On average, 19% of pupil diameter data per time interval were excluded from the analysis because missing eye gaze data made up > 50% of the samples. Moreover, outliers (deviating more than 3 *SD*s from the average diameter of a given participant during a particular time interval) were eliminated (on average, 0.8% of the remaining pupil diameter data per time interval).

### Dependent variables

One dependent variable that measured attention orientation during the visual search task consisted of participants’ RTs for correct responses (in ms); errors comprised ~ 5.5% of responses. The dependent eye tracking variables during the visual search task consisted of two components: attention orientation was measured by the time to first hit on the target (in ms; note that it was possible to detect the target in the visual search task without performing a saccade, which is why we cannot rule out effects of covert attention that might have interfered with this measure of attention orientation; however, this should only have weakened the effects of interest in our study) and attention maintenance was measured by the percentage of gazing at the target half a second after the first hit (in% of overall looking at the screen). Hits were defined as gaze points on the area of interest, which consisted of the target picture and 10% of the picture size added to each side. The employed measures and time intervals are commonly used in eye tracking research [46,47]. Trials in which participants did not gaze at the target at all were excluded from eye tracking analyses (an additional ~ 4.6% of all trials). Peripheral attention to target stimuli possibly led to these trials in which participants responded correctly even though they did not hit the target. For the percentage of gaze analysis, ~ 7.4% of all trials were additionally excluded because participants did not hit the target within the first 2000 ms of the presentation of the visual search task (and therefore the time spanning half a second after first hit would have exceeded the presentation of stimuli). In addition, ~ 2.2% of all trials were excluded due to missing eye gaze data of greater than 40% of the sample (mostly due to eye blinks).

### Data analysis

We hypothesized that (1) gain and loss cues elicit a stronger affective response, demonstrated by a larger increase in pupil diameter, than do ambiguous cues that serve as a control cue and should not contain a specific affective dimension (manipulation check). To test this hypothesis, we conducted a 3 × 5 analysis of variance (ANOVA) with the within-subject factors expectancy (gain cue [gain 90%], loss cue [loss 90%], ambiguous cue [gain loss 50%/loss gain 50%]) and time (0–0.5 s, 0.5–1 s, 1–1.5 s, 1.5–2 s, 2–2.5 s) on the pupil diameter change data. Our hypothesis should be reflected in a significant main effect of expectancy cue, as well as in a significant expectancy cue × time interaction. Significant main effects of expectancy cue and significant interactions of expectancy cue and time were further investigated by post-hoc pairwise comparisons.

Moreover, we hypothesized that (2a) gain cues, rather than loss cues, enhance attention to gain targets and (2b) loss cues, rather than gain cues, enhance attention to loss targets (cue congruency hypothesis). In addition, we predicted that (2c) gain cues enhance attention to gain targets rather than loss targets and (2d) loss cues enhance attention to loss targets rather than gain targets (target congruency hypothesis). To test these hypotheses, we conducted a 3 × 2 ANOVA with the within-subject factors expectancy (gain cue [gain 90%], loss cue [loss 90%], ambiguous cue [gain loss 50%/loss gain 50%]), and target (gain, loss) on RTs, the time to first hit on the target (attention orientation), and percentage of gazing at the target half a second after the first hit (attention maintenance). We also performed analyses on logarithmic RTs and excluded outliers (± 3 *SD*s from individual average RT). However, the effects observed in the current study were not affected by these data transformations. Therefore, only the results for the original RT data are described. Ambiguous cues that served as a control condition with maximum uncertainty in our experiment were included as an anchor in the analyses. If true, our hypotheses should be reflected in a significant interaction of the expectancy cue and target. Significant interaction effects were further investigated by post-hoc (Sidak corrected) pairwise comparisons. An α-level of .05 (two-tailed) was applied to all analyses (unless otherwise specified). Reported effect sizes are partial η^2^ and noted as η^2^_p_. If the sphericity assumption was violated, Greenhouse-Geisser corrected values are reported.

Furthermore, we hypothesized that (3) optimistic expectancies guide attention more toward gain targets compared with loss targets than pessimistic expectancies guide attention toward loss targets compared with gain targets (optimism robustness hypothesis). Therefore, two difference scores between four of our experimental conditions were computed:

$$\begin{array}{l} Diff_{GainCue}=\left[Gain\;cue,\;loss\;target\right]–\left[Gain\;cue,\;gain\;target\right]\\ Diff_{LossCue}=\left[Loss\;cue,\;gain\;target\right]–\left[Loss\;cue,\;loss\;target\right] \end{array}$$

We anticipated larger difference scores for optimistic expectancies than for pessimistic expectancies (Diff_GainCue_ > Diff_LossCue_) for the RTs and the time to first hit (attention orientation). We anticipated smaller difference scores for optimistic expectancies than for pessimistic expectancies (Diff_GainCue_ < Diff_LossCue_) for the percentage of looking at the target half a second after the first hit (attention maintenance). The last measure was expected to show negative difference scores because it was inverted to the RTs and time to first hit (i.e., enhanced attention results in shorter RTs and time to first hit but a larger percentage of looking at the target half a second after the first hit). To test the optimism robustness hypothesis, Diff_GainCue_ and Diff_LossCue_ were compared using pairwise *t*-tests with an α-level of .05 (one-tailed). The reported effect sizes are Cohen’s *d* and are denoted by *d*.

Last, we hypothesized that (4) optimism robustness scores in our experiment are positively associated with participants’ self-reported comparative optimism bias (cf. comparative optimism bias hypothesis). Comparative optimism bias was operationalized as overly optimistic expectancies about future life events for oneself compared with a person of the same age and gender measured by the COS [2]. Optimism robustness scores were computed with the following formula for the three attention measures (RTs, time to first hit, percentage of looking at target half a second after first hit):

$$Optimism\;Robustness\;Score=Diff_{GainCue}-Diff_{LossCue}$$

Because a large sample is needed to investigate inter-individual differences, we merged participants in the two studies (N = 63). A Pearson product-moment correlation was run to determine the relationship between participants’ mean score on the COS [2] and the optimism robustness score for each of the three measures of attention as revealed by our experiments. The α-level was set to .05 (one-tailed).

## Experiment 1: Results

The results of our experiments are reported in two sections–one devoted to analyses of the expectancy phase of our experiment (manipulation check: pupil diameter change) and another to analyses of the visual search phase (RTs, time to first hit the target and percentage of gazing at the target half a second after first hit revealed by eye tracking, and relation to comparative optimism bias). The mean values, standard errors and 95% confidence intervals for all experimental conditions from the described analyses are shown in S1 and S2 Tables. The difference scores related to the optimism robustness hypothesis are given in S3 Table. *F*-values and effect sizes are only reported for significant results of the ANOVAs. Statistical values for all effects (including non-significant results) can be found in S4 and S5 Tables. *P*-values for all post-hoc pairwise comparisons can be found in S6 and S7 Tables.

### Expectancy phase

#### Pupil diameter change

**Fig 2:**
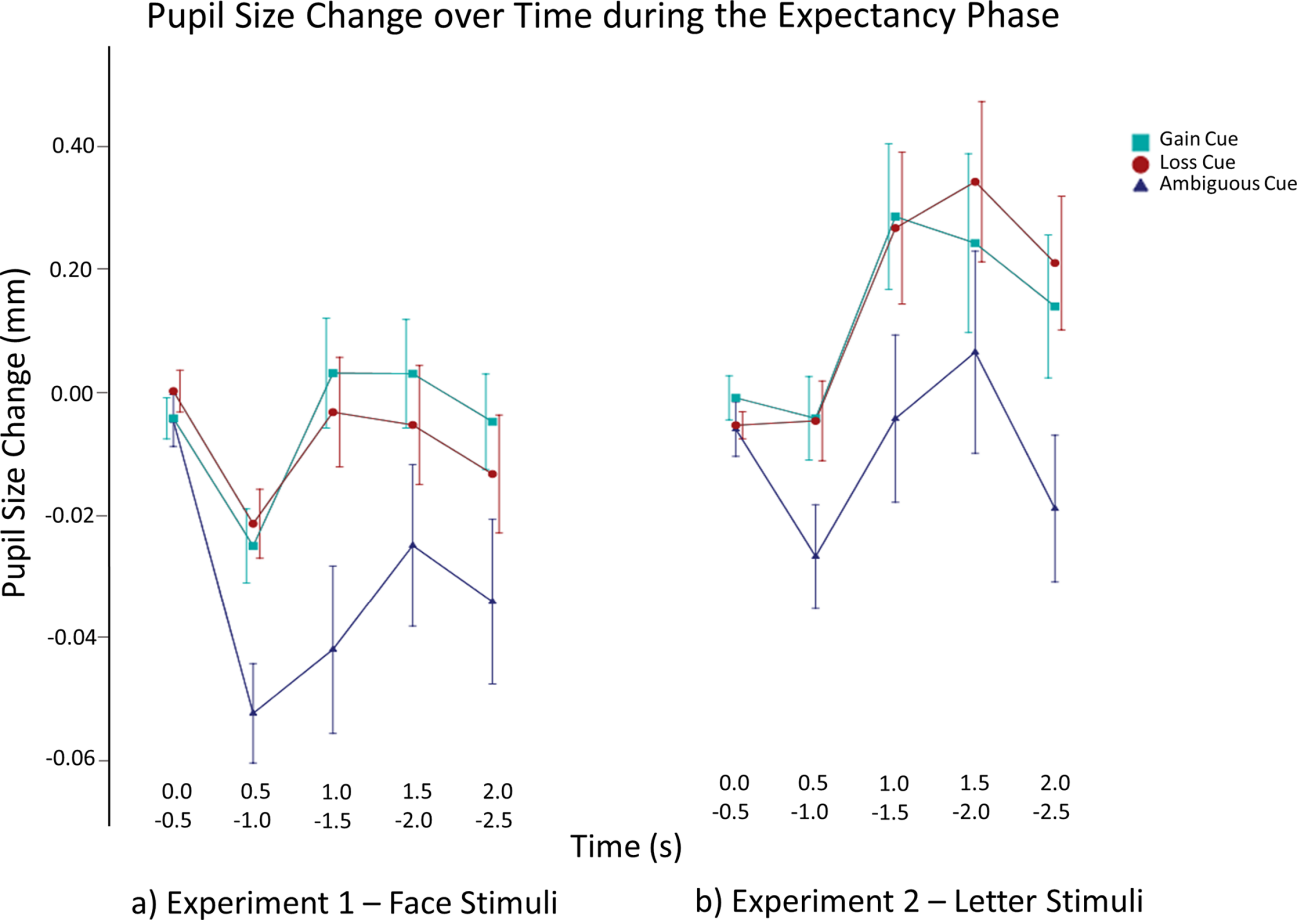
Pupil diameter change during the expectancy phase as a function of time and expectancy cue. Gain cue, loss cue, and ambiguous cue refer to the gain 90%, loss 90%, and gain loss [loss gain] 50% cues, respectively. The error bars depict standard errors.

Pupil diameter change during the presentation of expectancy cues is shown in Fig 2A. As predicted, the main effect of expectancy cue was significant, *F*_(1.44)_ = 11.854, *p* < .001, η^2^_p_ = .283. Gain and loss cues elicited a smaller decrease in pupil diameter than did ambiguous cues (gain vs. ambiguous cues: *p* = .001, loss vs. ambiguous cues: *p* = .007, as revealed by post-hoc pairwise comparisons). In addition, there was a significant main effect of time, *F*_(2.50)_ = 4.098, *p* = .029, η^2^_p_ = .120. Moreover, the predicted interaction expectancy × time was significant, *F*_(5.148)_ = 9.052, *p* < .001, η^2^_p_ = .232. Post-hoc pairwise comparisons showed that the differential effect of expectancy condition on pupil diameter change started between 0.5 and 1 s following the onset of the expectancy cues and remained stable until the end of the analysis interval. The pupil diameter increase was larger and the decrease was smaller for gain and loss cues than for ambiguous cues (0.5–1 s, 1–1.5 s, 1.5–2 s, 2–2.5 s: gain vs. ambiguous cues: *p*s = .005, < .001, = .002, = .004, respectively; loss vs. ambiguous cues: *p*s = .001, .001, .057, .072, respectively, as revealed by post-hoc pairwise comparisons).

### Visual search phase

#### Reaction times

**Fig 3:**
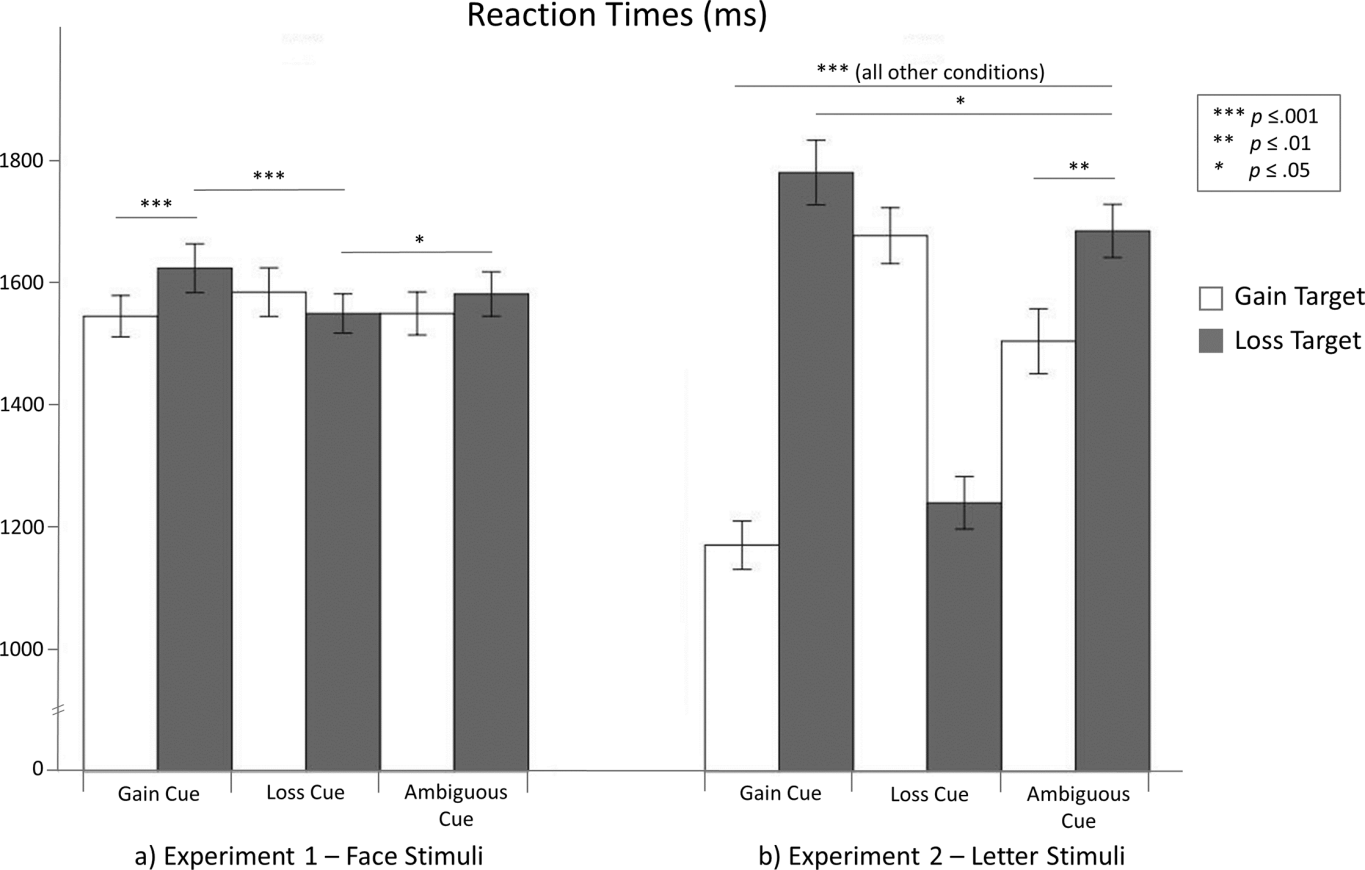
Reaction times. The error bars depict standard errors. The line labeled “all other conditions” indicates that all pairwise comparisons of the conditions encompassed by the line revealed highly significant differences (if not otherwise indicated).

The RTs are shown in Fig 3A. The RTs did not differ between gain and loss targets or between the three expectancy conditions, *p*s > .08. Notably, the predicted expectancy × target interaction was significant, *F*_(2.60)_ = 8.324, *p* = .001, η^2^_p_ = .217. As anticipated by our cue congruency hypothesis, participants reacted faster to loss targets when they expected to lose than when they expected to gain or had ambiguous expectancies (i.e., when neither optimistic nor pessimistic expectancies dominated; loss vs. gain cues: *p* = .001, loss vs. ambiguous cues: *p* = .041, as revealed by post-hoc pairwise comparisons). In line with our target congruency hypothesis, participants reacted faster to gain targets than to loss targets when they expected to gain (*p* = .001). Participants’ RTs did not differ significantly between any of the remaining conditions (all *p*s > .093). The optimism robustness hypothesis had to be rejected: Expecting to gain did not shorten RTs to gain targets compared with loss targets more than expecting to lose shortened RTs to loss targets compared with gain targets, even though there was a trend in the anticipated direction, *t*(30) = 1.602, *p* = .060, *d* = .381.

#### Eye tracking: Time to first hit the target

**Fig 4:**
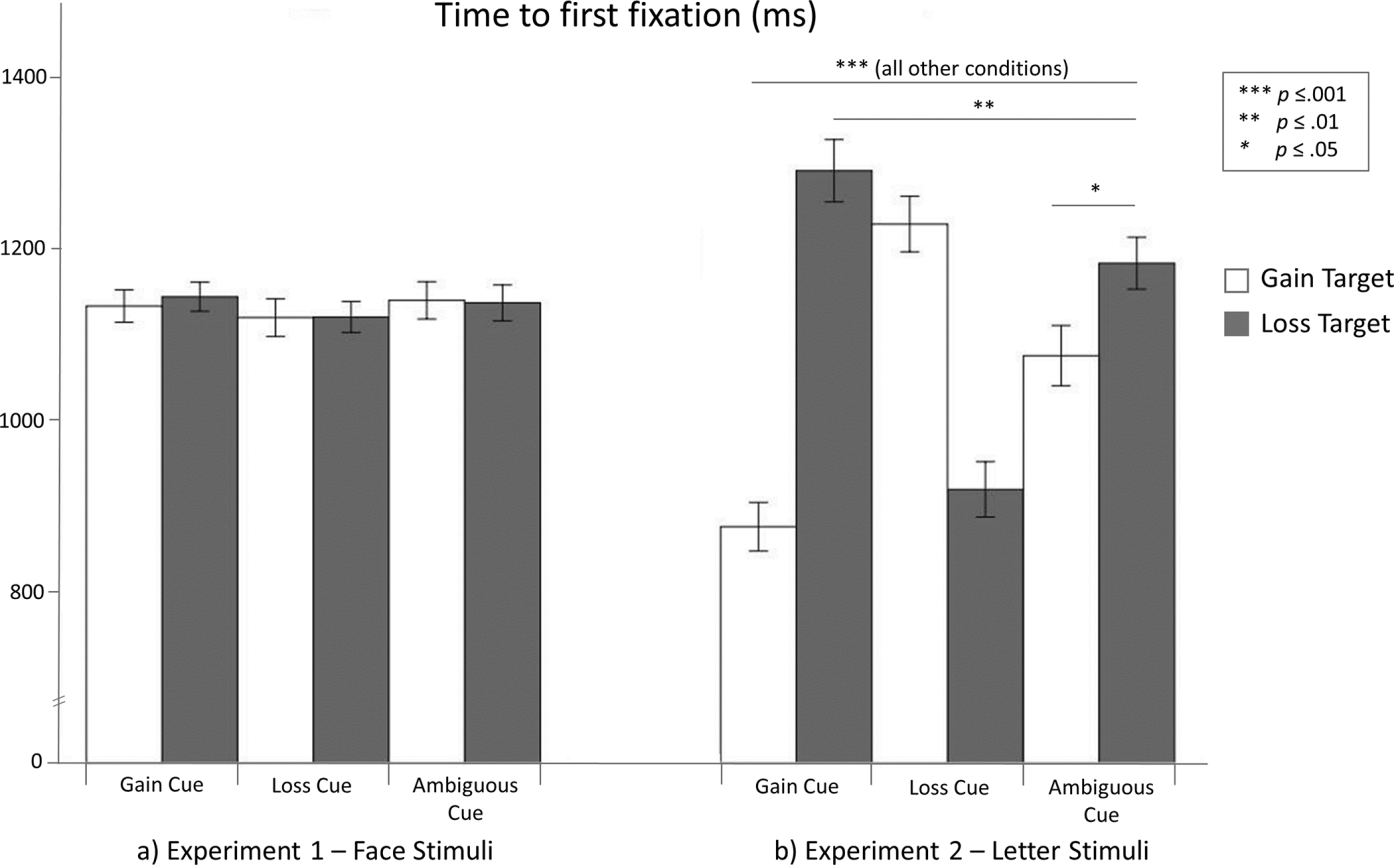
Time to first hit. The error bars depict standard errors. The line labeled “all other conditions” indicates that all pairwise comparisons of the conditions encompassed by the line revealed highly significant differences (if not otherwise indicated).

The time to first hit the target for the experimental conditions is shown in Fig 4A. The time to the first hit did not differ between gain and loss targets or between the three expectancy conditions, *p*s > .180. Contrary to our cue and target congruency hypotheses, the expectancy × target interaction was not significant, *p* = .849. Moreover, the optimism robustness hypothesis had to be rejected: Expecting to gain did not reduce the time to first hit gain targets compared with loss targets more than expecting to lose reduced the time to first hit loss targets compared with gain targets (*p* = .327).

#### Eye tracking: Percentage of gazing at the target half a second after the first hit

**Fig 5:**
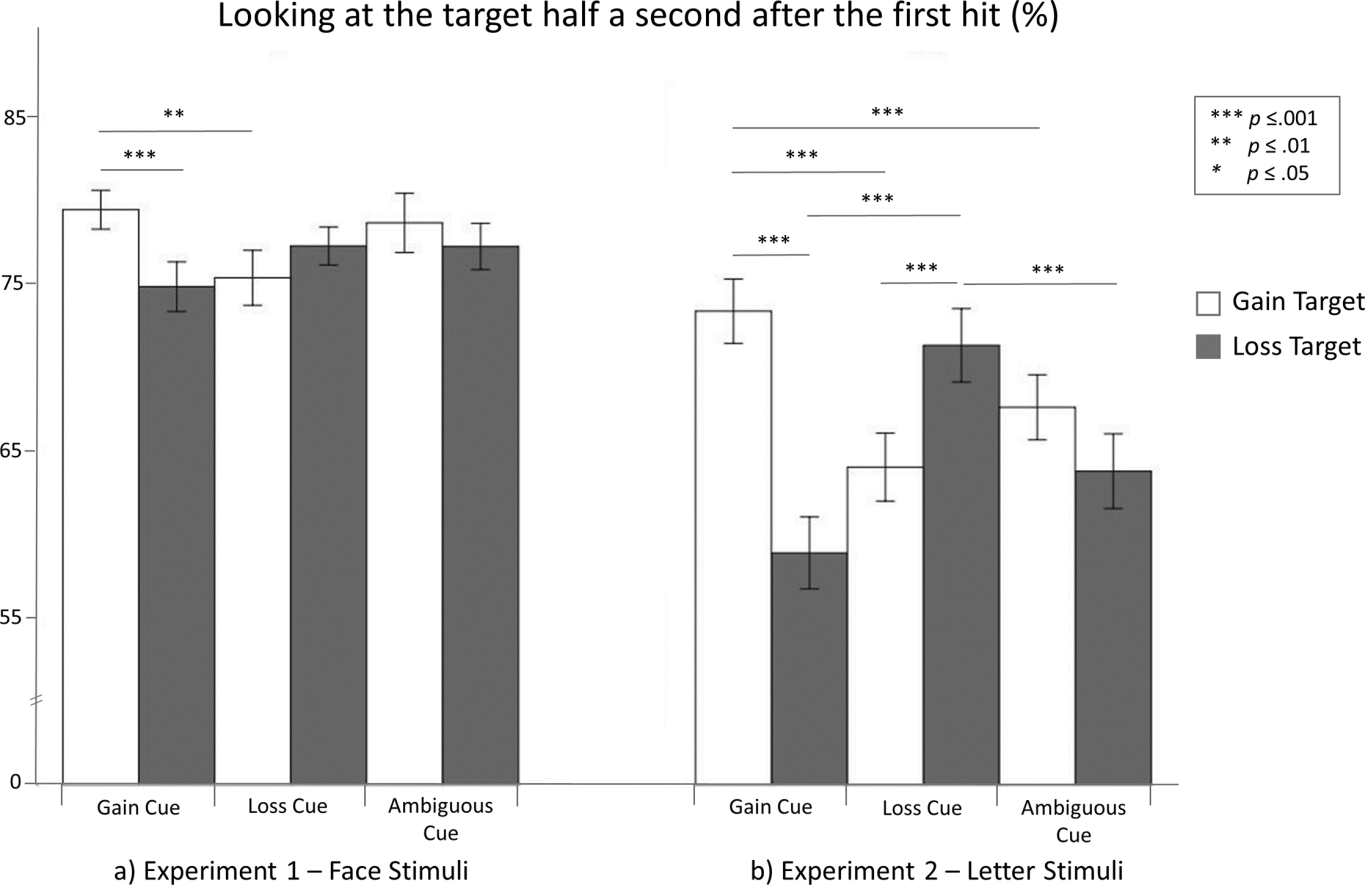
Percentage of gazing at the target half a second after the first hit. The error bars depict standard errors.

Fig 5A depicts the amount of time (in%) participants spent gazing at the target half a second after the first hit. Where participants gazed in this time span did not differ between gain and loss targets or between the three expectancy conditions, *p*s > .155. However, the predicted expectancy × target interaction was significant, *F*_(2.50)_ = 7.482, *p* = .002, η^2^_p_ = .200. In line with our cue congruency hypothesis, participants gazed more at gain targets within half a second after the first hit when they expected to gain than when they expected to lose (*p* = .009, as revealed by post-hoc pairwise comparisons). In line with our target congruency hypothesis, when participants expected to gain, they subsequently gazed longer at gain targets than at loss targets during the half second after the first hit (*p* = .001). The amount of time participants gazed at the target in the half second after the first hit did not differ among the remaining conditions (all *p*s > .066).

The optimism robustness hypothesis had to be rejected. However, expecting to gain showed a trend to increase the percentage of gazing at gain targets compared with loss targets more than expecting to lose increased the percentage of gazing at loss targets compared with gain targets half a second after the first hit, *t*(30) = 1.507, *p* = .071, *d* = .173.

#### Relation with comparative optimism bias

**Fig 6:**
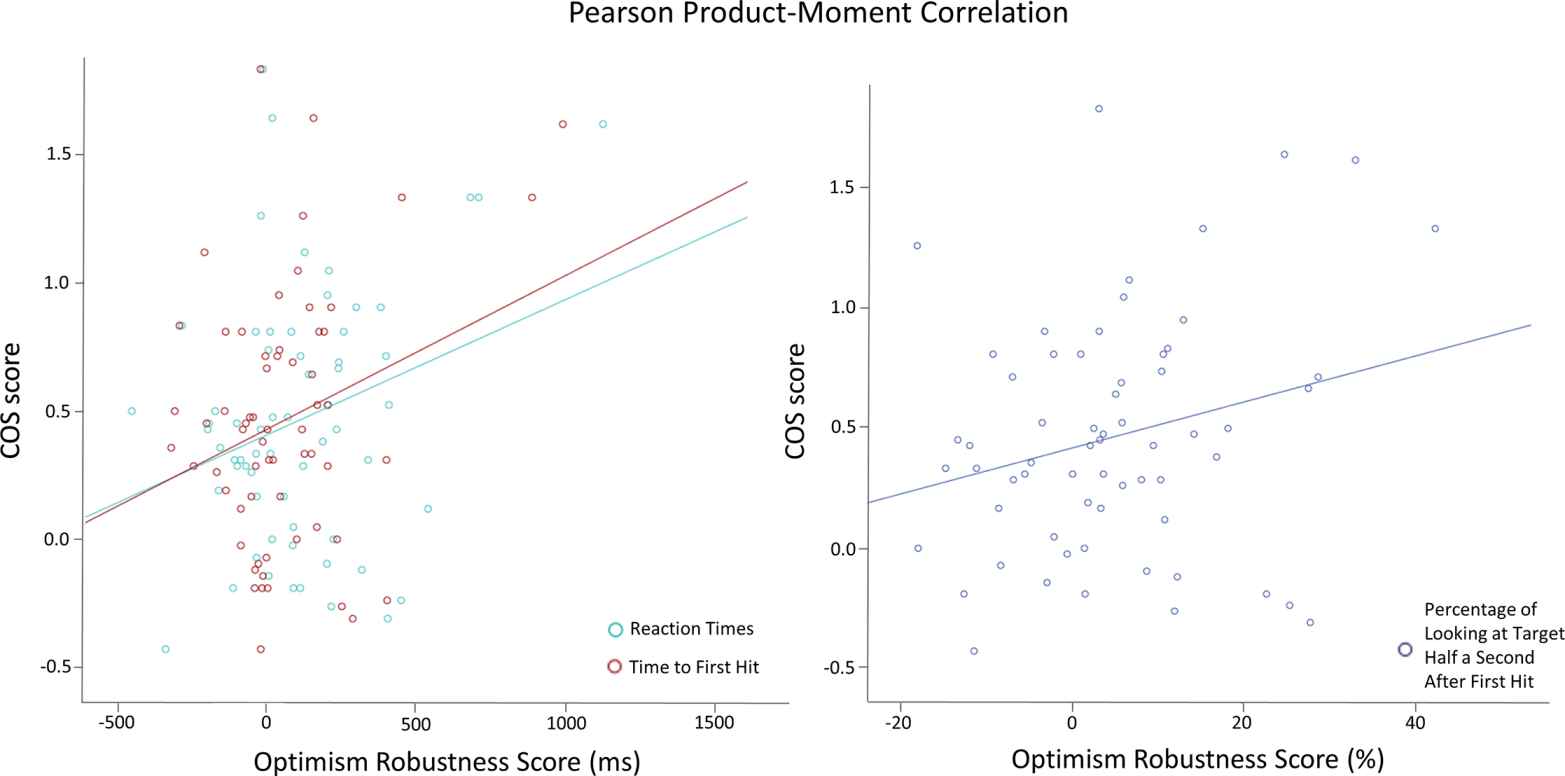
Correlation between participants’ mean COS score [2] and their optimism robustness scores revealed by the two experiments. Data of participants in Experiments 1 and 2 have been merged for this analysis in order to have a large enough sample size to investigate inter-individual differences. A score of zero represents no bias, a positive score represents a positivity bias, and a negative score represents a negativity bias in both measures.

Scatterplots of the correlations between optimism robustness scores and mean scores of the COS [2] for participants of both experiments are shown in Fig 6. As predicted in our comparative optimism bias hypothesis, there were significant weak, positive correlations between the mean score of the COS [2] and the optimism robustness score for all three measures of attention: RTs (*r*_*p*_ = .274, *p* = .015), time to first hit (*r*_*p*_ = .274, *p* = .015), and percentage of gazing at the target half a second after the first hit (*r*_*p*_ = .245, *p* = .027).

## Experiment 1: Discussion

As a manipulation check, we anticipated a larger increase in pupil diameter when participants were presented with gain or loss cues compared with ambiguous cues (control condition) because gain and loss cues were meant to elicit an affective response (i.e., optimistic and pessimistic expectancies). In line with our hypothesis, participants showed a significantly larger increase in pupil diameter for gain cues than for ambiguous cues during two of the analyzed time intervals (1–1.5 s and 1.5–2 s) in Experiment 1. In general, pupil diameter decreased in response to cue presentation and this decrease was significantly larger for ambiguous than for gain and loss cues. Therefore, the pupils were relatively more dilated during the presentation of gain and loss cues than during the presentation of ambiguous cues, indicating a stronger affective response elicited by gain and loss cues.

Moreover, in accordance with our predictions, optimistic expectancies modulated attention, as apparent in the RT and eye gaze data. Participants reacted faster to loss targets when they were pessimistic rather than optimistic and optimistic expectancies shortened RTs to gain targets compared with loss targets (attention orientation). No significant differences in RTs were detected between gain and loss targets when pessimistic or ambiguous expectancies were induced. Moreover, RTs to gain targets did not differ when optimistic expectancies were induced in comparison with pessimistic expectancies.

However, similar results were not seen for the time to first hit (attention orientation) in our eye tracking data. One possible explanation for this is that because of the numerous visual inputs participants received, they tried to obtain an overview in the beginning by gazing at all faces but then reacted faster to gain targets following gain cues even if they had only paid peripheral attention to those targets. However, more focused attention may subsequently have been diverted to gain targets following gain cues during later stages of attention. Consistent with such a view, the results for the percentage of gazing at the target half a second after the first hit (attention maintenance) were very similar to the effects seen in RTs. Participants looked more at gain targets when they had optimistic expectancies compared with pessimistic expectancies and optimistic expectancies made participants look more at gain targets compared with loss targets within a half second after the first hit at a target. Similar to the RTs, the percentage of looking at gain and loss targets half a second after the first hit did not differ when pessimistic or ambiguous expectancies were induced. Moreover, the percentage of looking at loss targets did not differ when optimistic expectancies were induced in comparison with pessimistic expectancies. In conclusion our cue and target congruency hypotheses could only be confirmed for optimistic expectancies, not for pessimistic expectancies.

Notably, although in our first hypothesis we had predicted that pessimistic expectancies guide attention toward punishment compared with reward, this result is congruent with our second hypothesis that optimistic expectancies have a stronger influence on subsequent attention to reward and punishment than pessimistic expectancies do. In line with our optimism robustness hypothesis, we found a small effect that optimistic expectancies shortened participants’ RTs to gain targets compared with loss targets more than pessimistic expectancies shortened participants’ RTs to loss targets compared with gain targets (attention orientation). A similar effect was seen in our eye tracking measure for attention maintenance. Optimistic expectancies made participants look more at gain targets compared with loss targets than pessimistic expectancies made participants look more at loss targets compared with gain targets half a second after the first hit. However, the trend for both effects was non-significant. Therefore, whether optimistic expectancies had a stronger effect on attention deployment to congruent confirming compared with disconfirming information than pessimistic expectancies was not clearly shown in our data and requires further investigation. In summary, our cue and target congruency hypotheses were only partly confirmed for attention orientation (RTs) and maintenance (percentage of gazing at target half a second after first hit) and the optimism robustness hypothesis was rejected for both attention measures.

As hypothesized, participants’ optimism robustness score for all three measures of attention revealed by our experiments is significantly positively correlated with the mean score of the COS [2]. This supports the idea that processes present in optimism bias also play a role in the robustness of optimistic expectancies and their influences on attention in our experiments.

Even though the results of Experiment 1 are promising, one problem with the stimuli used in this experiment is that happy and sad faces could not be assigned to be gain or loss targets differentially across participants. Happy faces always have a positive valence and sad faces always have a negative valence and it would not have been meaningful to tell participants they lose money when seeing a happy face. These salient stimulus-specific attributes could have differentially influenced attention deployment. For instance, in everyday life, we have repeatedly learned that a happy face indicates important emotional information (e.g., a smiling doctor telling us we are completely healthy or a happy supervisor complementing us on our work), making happy faces particularly salient stimuli that might be processed preferably regardless of the context in which they are presented. Therefore, independently of assigning happy and sad faces as gain and loss targets in our experiment, the face stimuli might have captured participants’ attention differently, making them less prone to variations in expectancies. Thus, we conducted a second experiment to replicate our effects using non-social and inherently non-emotional stimuli.

## Experiment 2: Methods and materials

Experiment 2 was a replication of Experiment 1 with different stimuli. As both experiments were highly similar, we describe only the details that differ from Experiment 1. If not otherwise indicated, the procedures were identical to Experiment 1.

### Participants

Thirty-two healthy psychology students (7 male, age: *M* = 22.19 years; *SD* = 3.00 years; range = 19–36 years) who had not participated in Experiment 1 were recruited via the participant pool at the University of Bern and took part in this RT and eye tracking study.

### Stimuli

Visual search task (attention): The stimuli consisted of a green and a red “L” and a green and a red “T”. The green “L” and the red “T” served as target stimuli and the red “L”s and green “T”s served as distractor stimuli. In each trial, eight red and green “L”s and “T”s were shown on a white background on a circle around the position where the fixation cross was presented. There was an equal probability for the single green “L” or the single red “T” to appear in any of the eight different locations on the circle. The participants’ task was to find the deviant target letter (green “L” or red “T”) among seven neutral distractor letters (red “L”s and green “T”s). In contrast to Experiment 1, in which the stimuli in the visual search array had to be compared using a rather complex attribute comprising many different features (emotional facial expression), the stimuli in Experiment 2 only had to be compared using two clearly separable features (color and shape). However, because the emotional face stimuli used in Experiment 1 are highly familiar and overlearned in everyday life, they may generally produce a stronger pop-out effect among neutral distractor faces than the letter stimuli used in Experiment 2.

### Experimental procedure

The procedure was identical to that of Experiment 1 (Fig 1, bottom). The only difference was that letters were presented as stimuli in the visual search task instead of faces. For half of the participants, the green “L” represented gain (loss) and the red “T” represented gain (loss) for the other half.

### Manipulation check

Three participants were excluded from pupil diameter change analysis because on average, more than 50% of their trials per time interval had to be excluded because of too much missing eye gaze data. On average, we excluded 21.7% of pupil diameter data per time interval from the analysis because missing eye gaze data made up > 50% of the samples. In addition, outliers (deviating more than 3 *SD*s from the average diameter of a given participant during a particular time interval) were eliminated (on average 0.9% of the remaining pupil diameter data per time interval).

### Dependent variables

Errors comprised ~ 7.7% of responses and were excluded from the RT analysis. For the eye tracking analyses, ~ 13.2% of trials were additionally excluded because participants did not hit the target. For the percentage of gaze analysis ~ 4.5% of trials were additionally excluded because participants did not hit the target within the first 2000 ms of the presentation of the visual search task. Additionally, ~ 4.3% of trials were excluded because missing eye gaze data comprised > 40% of the samples.

## Experiment 2: Results

### Expectancy phase

#### Pupil diameter change

Pupil diameter change during the presentation of expectancy cues is shown in Fig 2B. As predicted, the main effect of expectancy cue was significant, *F*_(2.56)_ = 12.438, *p* < .001, η^2^_p_ = .308. As anticipated, gain and loss cues elicited a larger pupil diameter increase than did ambiguous cues (gain vs. ambiguous cues: *p* < .001, loss vs. ambiguous cues: *p* = .001, as revealed by post-hoc pairwise comparisons). In addition, there was a significant main effect of time, *F*_(2.43)_ = 4.284, *p* = .029, η^2^_p_ = .133.

Moreover, the predicted interaction expectancy × time was significant, *F*_(4.120)_ = 4.988, *p* = .001, η^2^_p_ = .151. Post-hoc pairwise comparisons showed that the differential effect of expectancy condition on pupil diameter change started between 1 and 1.5 s (0.5–1 s for loss vs. ambiguous cues) following the onset of the expectancy cues and remained stable until the end of the analysis interval. As anticipated, pupil diameter increase was larger for gain and loss cues than for ambiguous cues (0.5–1 s, 1–1.5 s, 1.5–2 s, 2–2.5 s: gain vs. ambiguous cues: *p*s = .080, < .001, = .009, < .001, respectively; loss vs. ambiguous cues: *p*s = .005, = .005, = .005, < .001, respectively).

### Visual search phase

#### Reaction times

The RTs are shown in Fig 3B. Participants reacted faster to gain targets than to loss targets, showing a main effect of target, *F*_(1.31)_ = 12.582, *p* = .001, η^2^_p_ = .289. Moreover, participants reacted faster when they expected to gain or lose than when they had ambiguous expectancies, showing a main effect of expectancy, *F*_(2.46)_ = 28.227, *p* < .001, η^2^_p_ = .477. In addition, the predicted expectancy × target interaction was significant, *F*_(1.43)_ = 79.723, *p* < .001, η^2^_p_ = .720.

In accordance with our cue congruency hypothesis, participants reacted faster to gain targets when they expected to gain than when they expected to lose or had ambiguous expectancies (gain vs. loss cues: *p* < .001, gain vs. ambiguous cues: *p* < .001, as revealed by post-hoc pairwise comparisons). Moreover, participants reacted faster to gain targets when they had ambiguous expectancies than when they expected to lose (*p* < .001). Participants reacted faster to loss targets when they expected to lose than when they expected to gain or had ambiguous expectancies (loss vs. gain cues: *p* < .001, loss vs. ambiguous cues: *p* < .001). In addition, participants reacted faster to loss targets when they had ambiguous expectancies than when they expected to gain (*p* = .015).

As predicted by our target congruency hypothesis, when participants expected to gain, they reacted faster to gain targets rather than loss targets (*p* < .001); when they expected to lose they reacted faster to loss targets rather than gain targets (*p* < .001); and when they had ambiguous expectancies, they reacted faster to gain targets rather than loss targets (*p* = .002). The last effect is consistent with the idea of an attention bias for positive stimuli. Moreover, in line with our optimism robustness hypothesis, expecting to gain shortened RTs to gain targets compared with loss targets more than expecting to lose shortened RTs to loss targets compared with gain targets, *t*(31) = 3.019, *p* = .003, *d* = .501.

#### Eye tracking: Time to first hit the target

The time to first hit the target results mostly mirror the RT results and are shown in Fig 4B. Participants took less time to first hit gain targets compared with loss targets, showing a main effect of target, *F*_(1.31)_ = 7.247, *p* = .011, η^2^_p_ = .189. Moreover, they took less time to hit the target when they expected to lose than when they had ambiguous expectancies, showing a main effect of expectancy, *F*_(2.62)_ = 4.918, *p* = .010, η^2^_p_ = .137. Notably, the predicted expectancy × target interaction was significant, *F*_(2.49)_ = 72.432, *p* < .001, η^2^_p_ = .700.

In line with our cue congruency hypothesis, participants first hit gain targets faster when they expected to gain than when they expected to lose or had ambiguous expectancies (gain vs. loss cues: *p* < .001, gain vs. ambiguous cues: *p* < .001, as revealed by post-hoc pairwise comparisons). Moreover, participants hit gain targets faster when they had ambiguous expectancies than when they expected to lose (*p* < .001). Furthermore, as anticipated, participants hit loss targets faster when they expected to lose than when they expected to gain or had ambiguous expectancies (loss vs. gain cues: *p* < .001, loss vs. ambiguous cues: *p* < .001). In addition, participants hit loss targets faster when they had ambiguous expectancies than when they expected to gain (*p* = .003).

As predicted by our target congruency hypothesis, when participants expected to gain, they hit faster at gain targets rather than loss targets (*p* < .001) and when they expected to lose they hit faster at loss targets rather than gain targets (*p* < .001). When participants had ambiguous expectancies, they hit faster at gain targets rather than loss targets (*p* = .019), in agreement with the idea of an attention bias for positive stimuli. Finally, as stated in our optimism robustness hypothesis, expecting to gain reduced the time to hit at gain targets compared with loss targets more than expecting to lose reduced the time to hit at loss targets compared with gain targets, *t*(31) = 2.091, *p* = .023, *d* = .424.

#### Eye tracking: Percentage of gazing at the target half a second after the first hit

The amount of time (in%) participants spent gazing at the target half a second after the first hit is shown in Fig 5B. Participants gazed more at gain targets than loss targets in this time span, showing a main effect of target, *F*_(1.31)_ = 7.464, *p* = .010, η^2^_p_ = .194, but the amount of time participants spent gazing at the target did not differ among the three expectancy conditions (*p* = 224). Notably, the predicted expectancy × target interaction was significant, *F*_(2.50)_ = 31.007, *p* < .001, η^2^_p_ = .500.

As hypothesized by our cue congruency hypothesis, participants gazed more at gain targets within a half second after the first hit when they expected to gain than when they expected to lose or had ambiguous expectancies (gain vs. loss cues: *p* < .001, gain vs. ambiguous cues: *p* = .001, as revealed by post-hoc pairwise comparisons). Moreover, participants gazed more at loss targets within a half second after the first hit when they expected to lose than when they expected to gain or had ambiguous expectancies (loss vs. gain cues: *p* < .001, loss vs. ambiguous cues: *p* < .001). In line with our target congruency hypothesis, when participants expected to gain, they gazed more at gain targets than at loss targets within a half second after the first hit (*p* < .001). When participants expected to lose, they gazed more at loss targets than at gain targets within a half second after the first hit (*p* = .001). The amount of time participants spent gazing at the target a half second after the first hit did not differ among the remaining conditions (all *p*s > .063).

Finally, consistent with our optimism robustness hypothesis, expecting to gain increased percentage of gazing at gain targets compared with loss targets half a second after the first hit more than expecting to lose increased percentage of gazing at loss targets compared with gain targets half a second after the first hit, *t*(31) = 2.713, *p* = .006, *d* = .595.

## Experiment 2: Discussion

As hypothesized, a larger pupil diameter increase was evoked by gain and loss cues than by ambiguous cues in Experiment 2. This indicates that gain and loss cues elicited an affective response in our participants that can be attributed to the induction of optimistic and pessimistic expectancies, whereas ambiguous cues did not (manipulation check). Thus, differential effects of attention in our experiment can be attributed to the induction of optimistic and pessimistic expectancies.

In accordance with our predictions, the expectancies in Experiment 2 modulated attention deployment, as apparent in the RT and eye gaze data. Participants reacted faster to gain and loss targets when congruent expectancies were induced compared with incongruent expectancies. Furthermore, optimistic and pessimistic expectancies shortened RTs to congruent targets compared with incongruent targets (attention orientation). In the eye gaze data, the same effects were observed for the time to first hit the target (attention orientation) and the percentage of looking at the target half a second after the first hit (attention maintenance).

In line with the idea of a general attention bias to positive stimuli, participants payed more attention to gain compared with loss targets when ambiguous expectancies were induced. This attention bias could be explained by a natural Pavlovian tendency to approach reward stimuli. Research has shown that approaching (i.e., initiating a response to) punishment is more difficult than approaching reward [48,49]. Therefore, a Pavlovian facilitation to approach reward could make people pay more attention to gain compared with loss targets when having ambiguous expectancies.

In contrast to Experiment 1, the optimism robustness hypothesis was clearly supported: Optimistic expectancies biased participants’ attention more strongly toward gain targets in comparison with loss targets than pessimistic expectancies biased participants’ attention toward loss targets in comparison with gain targets, as shown by the RTs, time to first hit, and percentage of gazing at the target half a second after the first hit. The described Pavlovian tendency to approach reward but not punishment information could also represent an underlying mechanism of this optimism robustness effect because it explains why it might be more difficult to pay attention to loss targets when expecting to gain than to gain targets when expecting to lose. In conclusion, our cue and target congruency hypotheses and our optimism robustness hypothesis were confirmed for both attention orientation (RTs, time to first hit) and attention maintenance (percentage of gazing at the target half a second after the first hit).

## General discussion

Affective states that can be attributed to optimistic and pessimistic expectancies were successfully induced in the experiments reported here. Both experiments demonstrate that optimistic expectancies guide attention toward positive compared with negative stimuli. This was revealed in the RTs and eye gaze behavior during the visual search task in Experiment 1 for emotional face stimuli (except for the time to first hit) and in Experiment 2 for non-social letter stimuli. Moreover, in Experiment 2 we clearly demonstrated that pessimistic expectancies guide attention toward negative compared with positive stimuli. As predicted, optimistic expectancies had a stronger influence on attention deployment than pessimistic expectancies–shown by small-to-medium effects in the RT analyses of both experiments and the eye tracking analyses of Experiment 2. Moreover, this stronger influence of optimistic than pessimistic expectancies on attention was positively associated with individual differences in self-reported comparative optimism bias [2].

Modulation of attention by expectancy cues is in line with predictive coding theory, which states that humans create a mental template while expecting certain outcomes in their future and compare sensory information with this template [26,27]. Furthermore, our findings correspond to empirical work on the interplay between expectancies and attention deployment to neutral stimuli [23–25].

In both studies, we show that optimistic expectancies guide attention toward positive in contrast to negative stimuli, a finding that is in line with Peters and colleagues´ (2015) results, even though different methods to induce optimism were used. Peters and colleagues (2015) showed that participants whose state optimism was increased by the Best Possible Self Manipulation (and those whose state optimism had unexpectedly increased by a presumably neutral control manipulation) gazed less at angry faces and more at joyful faces; we showed that optimism induced by cues signaling reward biased participants’ attention toward rewarding compared with punishing stimuli (apparent in the RTs and eye gaze behavior). Therefore, inducing state optimism in the beginning of an experiment or inducing optimistic expectancies through cues on a trial-to-trial basis successfully bias subsequent attention deployment. Notably, Peters and colleagues (2015) could only show rather weak effects of state optimism on attention in post-hoc analyses on alternatively created experimental groups whereas we demonstrated much stronger effects of optimistic expectancies on attention to reward and replicated the effects using non-social stimuli.

In addition to replicating results that show optimistic expectancies guide attention toward reward in contrast to punishment, in our second experiment, we demonstrated that pessimistic expectancies guide attention toward stimuli signaling punishment in contrast to stimuli signaling reward. Notably, this effect was only present when non-social letter stimuli were used. This finding initially arose in Experiment 2 (which generally led to stronger effects), which appears to be counterintuitive as social face stimuli would better represent real life situations in which expectancies rely on information with an intrinsic affective meaning. A possible explanation lies in participants’ answers to the post-hoc questionnaire about the experiments. Participants in Experiment 2 reported expectancy cues to be more helpful and important for the subsequent visual search task than participants in Experiment 1 (see S1 Analysis). It is conceivable that the search task in Experiment 2 was simply more difficult because letter target stimuli stood out less among distractors than the face stimuli did in Experiment 1. Therefore, participants probably had to rely more strongly on the information given during the expectancy phase of the experiment. However, in some conditions, the RTs in Experiment 1 were longer than those in Experiment 2. Thus, it is also possible that social stimuli captured attention to a greater extent (which is likely due to the stimuli’s social interaction significance; potentially influential factors: emotional display signaling action intent, attractiveness, or gender). This implies that participants could withdraw attention from letters more easily than from faces. Because different participants were included in Experiments 1 and 2, it is difficult to draw final conclusions in this respect.

In both experiments, optimistic expectancies had a stronger influence on attention deployment than did pessimistic expectancies and this asymmetry in attention deployment was positively related to participants’ self-reported optimism bias. Whereas in Experiment 1 only optimistic expectancies influenced subsequent attention to rewarding and punishing stimuli, in Experiment 2 both optimistic and pessimistic expectancies influenced attention but the effect was stronger for optimistic than for pessimistic expectancies (optimism robustness hypothesis). This robustness of optimistic expectancies was present in measures of attention orientation and attention maintenance. Therefore, both more automatic and more controlled or strategic types of stimulus processing during the different stages of attention were strongly influenced by optimistic expectancies. This finding underscores the outstanding relevance of optimism in determining attention processes that rely on very different mechanisms (such as salience detection during attention orientation and emotion regulation during attention maintenance; see [50] for details on the mechanisms underlying different stages of attention bias).

Emotion regulation goals may explain why optimistic expectancies influenced attention more than pessimistic expectancies in our experiments: First, pessimistic expectancies may have been overridden (especially when stimuli were processed in a more controlled manner during attention maintenance [50]). In this case, strategic attention on rewarding stimuli (represented by no or smaller effects of pessimistic expectancies compared with optimistic expectancies on attention in our experiments) might be an emotion regulation strategy serving to maintain a positive affective state, which could ultimately provoke a positive feedback effect on initially positively biased expectancies, thereby generating and stabilizing optimism bias.

Second, it is conceivable that people with optimistic expectancies do not want to confront themselves with disconfirming negative evidence and thus avoid attending to stimuli signaling punishment, enhancing attention for rewarding evidence. In this case, avoidance of punishing stimuli with coexistent attention on rewarding stimuli following optimistic expectancies represents an emotion regulation strategy that maintains optimism bias. As explained in the introduction, optimism bias is primarily viewed as a protective mechanism and people are highly motivated to remain optimistic even considering contradictory information [1,3,9].

Observations from our two studies imply that optimism bias and attention bias are strongly interrelated with dynamic bi-directional influences between each other that might vigorously strengthen both biases in the long run. Notably, our data might elucidate *why* people maintain their overly optimistic expectancies even when confronted with disconfirming information whereas they overcome pessimistic expectancies [9]. Attention processes apparently play a crucial role in this highly interesting phenomenon in optimism bias. As seen in our experiments, people pay less attention to disconfirming punishing feedback (“bad news”; compared with rewarding feedback) when they are optimistic than to disconfirming rewarding feedback ("good news"; compared with punishing feedback) when they are pessimistic (optimism robustness hypothesis). This asymmetry in attention deployment can explain why people update their expectancies when receiving good news but not when receiving bad news. Bad news might not be processed as deeply as good news, resulting in selective updating of expectancies when good news is received.

To strengthen this interpretation of our data, we performed additional analyses on the evolution in RTs over the time course of our experiment (S2 Analysis). When participants were confronted with disconfirming rewarding feedback while they were pessimistic, they adapted their orientation of attention quite rapidly over the course of the experiment, as shown by faster RTs in the second block of the experiment (steep learning curve). In contrast, when participants were confronted with disconfirming punishing feedback while they were optimistic, they adapted their orientation of attention rather slowly, as shown by faster RTs only in the third and fourth blocks of the experiment (flat learning curve). Slower learning regarding necessary attentional switching when being optimistic might also be related to updated expectancies over the course of the experiment. In conclusion, our novel findings suggest an underlying cognitive (i.e., attention-related) mechanism for asymmetric updating of expectancies, a crucial phenomenon implicated in the maintenance of optimism bias, with direct implications for mental health [3,4,9].

Some methodological features of this work might limit the conclusions that can be drawn about how optimistic and pessimistic expectancies influence attention deployment. First, most participants learned that gain and loss cues in our studies did not really represent a 90% chance of gaining or losing. This might have weakened the influence of expectancy cues on participants. In our design, we had to reduce the actual chances of gaining or losing to obtain enough incongruent trials for data analysis. However, several important considerations show that the expectancy cues in our studies influenced participants: (a) in most of the announced “90% cue” trials (in 67% of these trials) the expectancy cues correctly predicted the subsequent target. Moreover, participants were informed that the computer randomly chose a target, possibly leading to probabilities that differed from the announced average value of 90%, thus reducing the likelihood that participants distrusted the cues; (b) past experiments have shown that instructions about proportions can be sufficient to produce corresponding behavioral effects [51] (Experiment 1), even if the given information does not represent the true proportions; and (c) even if participants did not consciously believe the expectancy cues, the (possibly unconscious) effect of these cues on attention was still visible in the RTs and eye gaze behavior, thereby demonstrating their effectiveness.

Second, one might argue that the expectancy cues used in our studies did not actually induce optimistic and pessimistic expectancies. It would be possible that participants only drew on the predictive cognitive information the cues entailed (i.e., which specific target to search for) when performing the visual search task. This would imply that the differences in attention deployment we found solely derive from the cue’s predictive information not from optimistic or pessimistic expectancies induced by the cues. However, such an interpretation of the data cannot explain the differential effect that gain compared with loss cues had on attention deployment in our studies (optimism robustness hypothesis) because the predictive cognitive information of the gain and loss cues was equal. Moreover, we demonstrate that participants with higher optimism bias scores as revealed by the COS [2] showed a stronger influence of gain compared with loss cues on their attention deployment. This implies that optimism and pessimism did indeed play a role in our experiments. Future studies might directly circumvent any doubts on whether the gain and loss cues in the present experimental design induce optimistic and pessimistic expectancies by adding a control condition containing a cue that is predictive of the target’s identity but is not associated with gains or losses.

Third, we told participants that there will always be a target present in the visual search array. Consequently, some participants in Experiment 1 reported in the post-experimental questionnaire to have first looked at one side of the screen and pressed the button for the opposite side if the target was not present on the first side without further looking for the target. However, this strategy was not reported to be used in Experiment 2, which led to greatly overlapping results, making it very likely that this search strategy did not actually influence results. Moreover, even though some participants noted the use of this strategy in Experiment 1, eye tracking data showed that our expectancy manipulation influenced attention maintenance, implying that even if participants reported that they only looked at one half of the search array, they possibly unconsciously gazed at the target. Notably, there is no reason to suspect that employment of such a strategy would have had differential implications for optimistic and pessimistic expectancies. Thus, none of the limiting features mentioned here should have greatly influenced the findings reported in this paper.

In general, the findings are an important contribution to a more nuanced view of the processes at the basis of optimism bias. Modulation of subsequent attention processes from optimism bias is especially interesting because of its beneficial effects for mental health. Knowing that biased attention processes underlie important phenomena (such as selective updating) in optimism bias, which in turn are related to mental health, could ultimately yield a better understanding of psychological disorders and possible treatments. For instance, in contrast to healthy people, patients suffering from depression update their expectancies in both optimistic and pessimistic directions [3]. Patients with depression do not display a positive attention bias but attend preferably to negative information [52]. Maladaptive attention processes caused by an absence of optimism bias and/or resulting in an absence of selective updating of expectancies in patients could be addressed by attention bias modification training or even training that targets both future expectancies and attention deployment [12,15,16]. This approach is particularly important as optimistic biases in expectancies and attention might mutually reinforce and strengthen each other over time. The relationship of optimistic expectancies and attention is also important in non-clinical settings. For instance, when having a rough day, people can engage in a form of emotion regulation that incorporates active attempts at thinking positively and being optimistic about the future, thereby automatically driving their focus of attention to rewarding things in their environment, which likely results in enhanced well-being.

In conclusion, our data show that optimistic and pessimistic expectancies influence how we see the world around us and which aspects of our environment we direct attention to. Optimistic expectancies appear to be very powerful in biasing our attention to rewarding information, which underscores the uniqueness of optimism-related processing in humans and might provide information on which cognitive mechanisms are essential for the benefits of optimism bias. This can be central for fostering individual well-being and mental health. As we have shown that being optimistic or pessimistic influences which parts of our environment we pay attention to, we agree with Charlie Chaplin’s famous words and know that we should look up and use all the optimism we can muster to ensure we see the beautiful rainbow.

## Supporting information

S1 Analysis Differences between participants’ answers to questionnaires in Experiments 1 and 2. (DOCX)

S2 Analysis Evolution of RTs over time. (DOCX)

S1 Fig RT development of incongruent conditions over four blocks. To simplify the graph, the remaining conditions are not depicted although statistics were run on all experimental conditions. The error bars depict standard errors.
(TIF)

S1 Table Mean values, standard errors, and 95% confidence intervals (CIs) of pupil diameter change for the three expectancy cue conditions during five analyzed 0.5-s time intervals following cue onset in Experiments 1 (N = 31) and 2 (N = 32). (DOCX)

S2 Table Mean values, standard errors, and 95% confidence intervals (CIs) of reaction times, time to first hit, and percentage of gazing at the target half a second after the first hit are summarized for all experimental conditions analyzed in Experiments 1 and 2. (DOCX)

S3 Table Difference scores of reaction times, time to first hit, and percentage of gazing at the target half a second after the first hit are summarized for Experiments 1 and 2. (DOCX)

S4 Table Statistical values from the 3 (expectancy: gain, loss, ambiguous) x 5 (time: 0–0.5 s, 0.5–1 s, 1–1.5 s, 1.5–2 s, 2–2.5 s) ANOVA are given for the pupil diameter change analysis from Experiments 1 and 2. Significant *p*-values are marked with an asterisk.
(DOCX)

S5 Table Statistical values from the 3 (expectancy: Gain, loss, ambiguous) x 2 (target: Gain, loss) ANOVA are given for the reaction time analysis, time to first hit analysis, and percentage of gazing at the target half a second after the first hit analysis in Experiments 1 and 2. Significant *p*-values are marked with an asterisk.
(DOCX)

S6 Table *P*-values from post-hoc pairwise *t*-tests (Sidak corrected) comparing pupil diameter change for the different expectancy cues during five 0.5-s time intervals following cue onset in Experiments 1 and 2. Significant *p*-values are marked with an asterisk.
(DOCX)

S7 Table *P*-values from post-hoc pairwise *t*-tests (Sidak corrected) comparing different experimental conditions are given for the reaction time analysis, time to first hit analysis, and percentage of gazing at the target half a second after the first hit analysis in Experiments 1 and 2. Significant *p*-values are marked with an asterisk.
(DOCX)

S8 Table Number (percentage) of participants answering “yes” and “no” to questions on the post-experimental questionnaire in Experiments 1 and 2. (DOCX)

S1 Data File Raw data of all described analyses. (XLSX)

## References

[1] Sharot T. The optimism bias. Curr. Biol. 2011; 21: R941–5. doi: 10.1016/j.cub.2011.10.030 22153158 10.1016/j.cub.2011.10.030

[2] Weinstein ND. Unrealistic optimism about future life events. J Pers So Psychol. 1980; 39: 806–820. doi: 10.1037//0022-3514.39.5.806

[3] Korn CW, Sharot T, Walter H, Heekeren HR, Dolan RJ. Depression is related to an absence of optimistically biased belief updating about future life events. Psychol Med. 2014; 44: 579–592. doi: 10.1017/S0033291713001074 23672737 10.1017/S0033291713001074 PMC3880066

[4] Garrett N, Sharot T, Faulkner P, Korn CW, Roiser JP, Dolan RJ. Losing the rose tinted glasses: neural substrates of unbiased belief updating in depression. Front Hum Neurosci. 2014; 8 doi: 10.3389/fnhum.2014.00639 25221492 10.3389/fnhum.2014.00639 PMC4147849

[5] Shepperd JA, Waters EA, Weinstein ND, Klein WMP. A Primer on Unrealistic Optimism. Curr Dir Psychol Sci. 2015; 24: 232–237. doi: 10.1177/0963721414568341 26089606 10.1177/0963721414568341 PMC4467896

[6] Armor DA, Taylor SE. Situated Optimism: Specific Outcome Expectancies and Self-Regulation. Adv Exp Soc Psychol. 1998; 30: 309–379.

[7] Dillard AJ, Midboe AM, Klein WMP. The Dark Side of Optimism: Unrealistic Optimism About Problems With Alcohol Predicts Subsequent Negative Event Experiences. Pers Soc Psychol Bull. 2009; 35: 1540–1550. doi: 10.1177/0146167209343124 19721102 10.1177/0146167209343124

[8] Weinstein ND, Marcus SE, Moser RP. Smokers' unrealistic optimism about their risk. Tob Control. 2005; 14: 55–59. doi: 10.1136/tc.2004.008375 15735301 10.1136/tc.2004.008375 PMC1747991

[9] Sharot T, Korn CW, Dolan RJ. How unrealistic optimism is maintained in the face of reality. Nat. Neurosci. 2011; 14: 1475–1479. doi: 10.1038/nn.2949 21983684 10.1038/nn.2949 PMC3204264

[10] Pool E, Brosch T, Delplanque S, Sander D. Attentional bias for positive emotional stimuli: A meta-analytic investigation. Psychol Bull. 2016; 142: 79–106. doi: 10.1037/bul0000026 26390266 10.1037/bul0000026

[11] Becker DV, Anderson US, Mortensen CR, Neufeld SL, Neel R. The face in the crowd effect unconfounded: happy faces, not angry faces, are more efficiently detected in single- and multiple-target visual search tasks. J Exp Psychol Gen. 2011; 140: 637–659. doi: 10.1037/a0024060 21744984 10.1037/a0024060

[12] Everaert J, Koster EHW, Derakshan N. The combined cognitive bias hypothesis in depression. Clin Psychol Rev. 2012; 32: 413–424. doi: 10.1016/j.cpr.2012.04.003 22681914 10.1016/j.cpr.2012.04.003

[13] Berridge KC, Kringelbach ML. Affective neuroscience of pleasure: reward in humans and animals. Psychopharmacology. 2008; 199: 457–480. doi: 10.1007/s00213-008-1099-6 18311558 10.1007/s00213-008-1099-6 PMC3004012

[14] Schultz W. Neural coding of basic reward terms of animal learning theory, game theory, microeconomics and behavioural ecology. Curr Opin Neurobiol. 2004; 14: 139–147. doi: 10.1016/j.conb.2004.03.017 15082317 10.1016/j.conb.2004.03.017

[15] Hirsch CR, Clark DM, Mathews A. Imagery and interpretations in social phobia: Support for the combined cognitive biases hypothesis. Behav Ther. 2006; 37: 223–236. doi: 10.1016/j.beth.2006.02.001 16942974 10.1016/j.beth.2006.02.001

[16] Aue T, Okon-Singer H. Expectancy biases in fear and anxiety and their link to biases in attention. Clin Psychol Rev. 2015; 42: 83–95. doi: 10.1016/j.cpr.2015.08.005 26379081 10.1016/j.cpr.2015.08.005

[17] Kress L, Aue T. The link between optimism bias and attention bias: A neurocognitive perspective. Neurosc Biobehav Rev. 2017; 80: 688–702. doi: 10.1016/j.neubiorev.2017.07.016 28780313 10.1016/j.neubiorev.2017.07.016

[18] Armstrong T, Olatunji BO. Eye tracking of attention in the affective disorders: A meta-analytic review and synthesis. Clin Psychol Rev. 2012; 32: 704–723. doi: 10.1016/j.cpr.2012.09.004 23059623 10.1016/j.cpr.2012.09.004 PMC3556338

[19] Caseras X, Garner M, Bradley BP, Mogg K. Biases in visual orienting to negative and positive scenes in dysphoria: An eye movement study. J Abnorm Psychol. 2007; 116: 491–497. doi: 10.1037/0021-843X.116.3.491 17696705 10.1037/0021-843X.116.3.491

[20] Mohanty A, Gitelman DR, Small DM, Mesulam MM. The spatial attention network interacts with limbic and monoaminergic systems to modulate motivation-induced attention shifts. Cereb Cortex. 2008; 18: 2604–2613. doi: 10.1093/cercor/bhn021 18308706 10.1093/cercor/bhn021 PMC2567423

[21] Hahn S, Gronlund SD. Top-down guidance in visual search for facial expressions. Psychon Bull Rev. 2007; 14: 159–165. 17546747 10.3758/bf03194044

[22] Williams MA, Moss SA, Bradshaw JL, Mattingley JB. Look at me, I'm smiling: Visual search for threatening and nonthreatening facial expressions. Vis Cogn. 2005; 12: 29–50. doi: 10.1080/13506280444000193

[23] Aue T, Guex R, Chauvigné LAS, Okon-Singer H. Varying expectancies and attention bias in phobic and non-phobic individuals. Front Hum Neurosci. 2013; 7: 418 doi: 10.3389/fnhum.2013.00418 23964219 10.3389/fnhum.2013.00418 PMC3737492

[24] Burra N, Kerzel D. Attentional capture during visual search is attenuated by target predictability: evidence from the N2pc, Pd, and topographic segmentation. Psychophysiology. 2013; 50: 422–430. doi: 10.1111/psyp.12019 23418888 10.1111/psyp.12019

[25] Aue T, Chauvigne LAS, Bristle M, Okon-Singer H, Guex R. Expectancy influences on attention to threat are only weak and transient: Behavioral and physiological evidence. Biol Psychol. 2016; 121: 173–186. doi: 10.1016/j.biopsycho.2016.07.006 27396748 10.1016/j.biopsycho.2016.07.006

[26] Summerfield C, Egner T, Greene M, Koechlin E, Mangels J, Hirsch J. Predictive codes for forthcoming perception in the frontal cortex. Science. 2006; 314: 1311–1314. doi: 10.1126/science.1132028 17124325 10.1126/science.1132028

[27] Zelano C, Mohanty A, Gottfried JA. Olfactory Predictive Codes and Stimulus Templates in Piriform Cortex. Neuron. 2011; 72: 178–187. doi: 10.1016/j.neuron.2011.08.010 21982378 10.1016/j.neuron.2011.08.010 PMC3190127

[28] Scheier MF, Carver CS, Bridges MW. Distinguishing optimism from neuroticism (and trait anxiety, self-mastery, and self-esteem): a reevaluation of the Life Orientation Test. J Pers Soc Psychol. 1994; 67: 1063–1078. 7815302 10.1037//0022-3514.67.6.1063

[29] Karademas EC, Kafetsios K, Sideridis GD. Optimism, self-efficacy and information processing of threat- and well-being-related stimuli. Stress Health. 2007; 23: 285–294. doi: 10.1002/smi.1147

[30] Segerstrom SC. Optimism and Attentional Bias for Negative and Positive Stimuli. Pers Soc Psychol Bull. 2001; 27: 1334–1343. doi: 10.1177/01461672012710009

[31] Isaacowitz DM. The gaze of the optimist. Pers Soc Psychol Bull. 2005; 31: 407–415. doi: 10.1177/0146167204271599 15657455 10.1177/0146167204271599

[32] Peters ML, Vieler JS, Lautenbacher S. Dispositional and induced optimism lead to attentional preference for faces displaying positive emotions. An eye-tracker study. J Posit Psychol. 2015: 1–12. doi: 10.1080/17439760.2015.1048816

[33] Bateson M. Optimistic and pessimistic biases. A primer for behavioural ecologists. Curr Opin Behav Sci. 2016; 12: 115–121. doi: 10.1016/j.cobeha.2016.09.013

[34] Bradley MM, Miccoli L, Escrig MA, Lang PJ. The pupil as a measure of emotional arousal and autonomic activation. Psychophysiology. 2008; 45: 602–607. doi: 10.1111/j.1469-8986.2008.00654.x 18282202 10.1111/j.1469-8986.2008.00654.x PMC3612940

[35] Craik FI. Levels of processing: Past, present … and future. Memory. 2002; 10: 305–318. doi: 10.1080/09658210244000135 12396643 10.1080/09658210244000135

[36] Carretie L, Martin-Loeches M, Hinojosa JA, Mercado F. Emotion and attention interaction studied through event-related potentials. J Cogn Neurosci. 2001; 13: 1109–1128. doi: 10.1162/089892901753294400 11784449 10.1162/089892901753294400

[37] Fashler SR, Katz J. More than meets the eye: visual attention biases in individuals reporting chronic pain. J Pain Res. 2014; 7: 557–570. doi: 10.2147/JPR.S67431 25285022 10.2147/JPR.S67431 PMC4181742

[38] Seymour B, Daw N, Dayan P, Singer T, Dolan R. Differential encoding of losses and gains in the human striatum. J Neurosci. 2007; 27: 4826–4831. doi: 10.1523/JNEUROSCI.0400-07.2007 17475790 10.1523/JNEUROSCI.0400-07.2007 PMC2630024

[39] Tottenham N, Borscheid A, Ellertsen K, Marcus DJ, Nelson CA. Categorization of facial expressions in children and adults: Establishing a larger stimulus set. J Cogn Neurosci. 2002: 74.

[40] Hanssen M. M., Vancleef L. M., & Peters M. L. What does it mean to be an optimist in an ambiguous world? Investigating negative versus positive interpretation patterns of optimists. submitted.

[41] Diener E, Emmons RA, Larsen RJ, Griffin S. The Satisfaction With Life Scale. J Pers Assess. 1985; 49: 71–75. doi: 10.1207/s15327752jpa4901_13 16367493 10.1207/s15327752jpa4901_13

[42] Watson D, Clark LA, Tellegen A. Development and validation of brief measures of positive and negative affect: The PANAS scales. J Pers Soc Psychol. 1988; 54: 1063–1070. 3397865 10.1037//0022-3514.54.6.1063

[43] Gross JJ, John OP. Individual differences in two emotion regulation processes: implications for affect, relationships, and well-being. J Pers Soc Psychol. 2003; 85: 348–362. 12916575 10.1037/0022-3514.85.2.348

[44] Carver CS, White TL. Behavioral inhibition, behavioral activation, and affective responses to impeding reward and punishment: The BIS/BAS Scales. J Pers Soc Psychol. 1994; 67: 319–333.

[45] Rammstedt B. The 10-Item Big Five Inventory—Norm values and investigation of sociodemographic effects based on a German population representative sample. Eur J Psychol Assess. 2007; 23: 193–201. doi: 10.1027/1015-5759.23.3.193

[46] Palanica A, Itier RJ. Searching for a perceived gaze direction using eye tracking. J Vis. 2011; 11 doi: 10.1167/11.2.19 21367758 10.1167/11.2.19 PMC3933318

[47] Wieser MJ, Pauli P, Weyers P, Alpers GW, Muhlberger A. Fear of negative evaluation and the hypervigilance-avoidance hypothesis: an eye-tracking study. J Neural Transm. 2009; 116: 717–723. doi: 10.1007/s00702-008-0101-0 18690409 10.1007/s00702-008-0101-0

[48] Guitart-Masip M, Fuentemilla L, Bach DR, Huys QJM, Dayan P, Dolan RJ, et al Action dominates valence in anticipatory representations in the human striatum and dopaminergic midbrain. J Neurosci. 2011; 31: 7867–7875. doi: 10.1523/JNEUROSCI.6376-10.2011 21613500 10.1523/JNEUROSCI.6376-10.2011 PMC3109549

[49] Guitart-Masip M, Duzel E, Dolan R, Dayan P. Action versus valence in decision making. Trends Cogn Sci. 2014; 18: 194–202. doi: 10.1016/j.tics.2014.01.003 24581556 10.1016/j.tics.2014.01.003 PMC3989998

[50] Cisler JM, Koster EHW. Mechanisms of attentional biases towards threat in anxiety disorders: An integrative review. Clin Psychol Rev. 2010; 30: 203–216. doi: 10.1016/j.cpr.2009.11.003 20005616 10.1016/j.cpr.2009.11.003 PMC2814889

[51] Entel O, Tzelgov J, Bereby-Meyer Y. Proportion congruency effects: instructions may be enough. Front Psychol. 2014; 5: 1108 doi: 10.3389/fpsyg.2014.01108 25339929 10.3389/fpsyg.2014.01108 PMC4186283

[52] Gotlib IH, Krasnoperova E, Yue DN, Joormann J. Attentional biases for negative interpersonal stimuli in clinical depression. J Abnorm Psychol. 2004; 113: 127–135. doi: 10.1037/0021-843X.113.1.127 10.1037/0021-843X.113.1.121 14992665

