# Supplementary material for: Seeing through rose-colored glasses: How optimistic expectancies guide visual attention
Source: PLoS One. 2018 Feb 21;13(2):e0193311. doi: 10.1371/journal.pone.0193311 (PMC5821386; doi:10.1371/journal.pone.0193311)
Supplement: S1 Analysis — (DOCX) [file pone.0193311.s001.docx]

**S1 Analysis. Differences between participants’ answers to questionnaires in Experiments 1 and 2.**

**Data Analysis**

The participants indicated if (a) they had paid attention to the expectancy cues, (b) the cues were important to prepare their answer, (c) the cues helped them to answer as quickly and correctly as possible, and (d) the cues influenced the difficulty to find the targets on a post-experimental questionnaire. To obtain information on whether participants’ experience of the task might have differed among the two experiments, the differences between participants’ answers (“yes” or “no”) on the post-experimental questionnaire in Experiments 1 and 2 were investigated with a *χ*^2^ test (df = 1). An α-level of .05 (two-tailed) was applied.

Moreover, participants completed the LOT-R [28] measuring trait optimism. To ensure that trait optimism did not differ between participants in the two experiments, the LOT-R scores [28] were compared between participants in Experiments 1 and 2 using a *t*-test for independent samples (two-tailed).

**Results**

In the post-experimental questionnaires of both Experiments 1 and 2, most participants reported that they paid attention to the expectancy cues, indicating that they followed the task instructions well (Experiment 1: 74 %, Experiment 2: 88 %). The answers to the post-experimental questionnaire are shown in S8 Table.

In contrast to Experiment 1, most participants in Experiment 2 reported that the expectancy cues presented in the beginning of each trial were important to prepare their reaction (*χ*^2^ = 19.693, *p* < .001), helped them to answer as quickly and correctly as possible (*χ*^2^ = 15.276, *p* < .001), and influenced how difficult it was to find a target (*χ*^2^ = 9.958, *p* = .002; see S8 Table for details). Trait optimism did not differ between participants of Experiments 1 and 2, *t*_(61)_ = .024, *p* = .981 (*M*_Exp 1_ = 22.710 and *M*_Exp 2_ = 22.688).

**Conclusion**

Even though participants in both experiments followed the task instructions, participants in Experiment 2 found the expectancy cues to be more helpful for the subsequent visual search task than participants in Experiment 1. This might show why the effects of expectancies on attention deployment were generally stronger in Experiment 2 than in Experiment 1. However, because different participants were included in Experiments 1 and 2, it is difficult to draw conclusions about the distinct mechanisms employed in the experiments.
