# Supplementary material for: Seeing through rose-colored glasses: How optimistic expectancies guide visual attention
Source: PLoS One. 2018 Feb 21;13(2):e0193311. doi: 10.1371/journal.pone.0193311 (PMC5821386; doi:10.1371/journal.pone.0193311)
Supplement: S2 Analysis — (DOCX) [file pone.0193311.s002.docx]

**S2 Analysis. Evolution of RTs over time.**

**Data Analysis**

To further examine our optimism robustness hypothesis, we analyzed the changes in RTs over time. The evolution of RTs in the two incongruent conditions (gain cue, loss target and loss cue, gain target) could reveal differential updating mechanisms when incongruent feedback is given, underscoring the robustness of optimistic expectancies in contrast to pessimistic expectancies. Therefore, time was added to the ANOVA as a within-subject factor. As a result, a 4 × 3 × 2 ANOVA with the within-subject factors time (block 1, block 2, block 3, and block 4), expectancy (gain cue [gain 90 %], loss cue [loss 90 %], ambiguous cue [gain loss 50 %/loss gain 50 %]), and target (gain, loss) was conducted on the RTs. The focus of this analysis was on the development of RTs in the two incongruent conditions (gain cue, loss target and loss cue, gain target), which are depicted in S1 Fig. Therefore, only post-hoc tests that reveal information on the development of these two conditions over time were reported.

**Results**

Experiment 1: Participants reacted faster in block 3 than in block 1, showing a main effect of time, *F*_(3.84)_ = 5.902, *p* = .001, η^2^_p_ = .174. There was a significant time × expectancy interaction, *F*_(6.168)_ = 3.211, *p* = .005, η^2^_p_ = .103, and a significant expectancy × target interaction, *F*_(2.56)_ = 6.497, *p* = .003, η^2^_p_ = .188. In addition, there was a trend for a time × expectancy × target interaction, *F*_(6.168)_ = 1.876, *p* = .088, η^2^_p_ = .063. Post-hoc pairwise comparisons showed that when participants expected to gain, they only reacted significantly faster to loss targets in block 3 compared with block 1 (*p* = .032). In contrast, when participants expected to lose, they reacted significantly faster to gain targets in blocks 2, 3, and 4 compared with block 1 (block 1 vs. block 2: *p* = .001, block 1 vs. block 3: *p* = .005, block 1 vs. block 4: *p* = .002).

Experiment 2: Participants reacted faster in blocks 2, 3, and 4 than in block 1 and faster in blocks 3 and 4 than in block 2, showing a main effect of time, *F*_(3.78)_ = 27.609, *p* < .001, η^2^_p_ = .471. Moreover, participants reacted faster when they expected to gain or lose than when they had ambiguous expectancies, showing a main effect of expectancy, *F*_(2.46)_ = 26.697, *p* < .001, η^2^_p_ = .463. Participants reacted faster to gain targets than to loss targets, showing a main effect of target, *F*_(1.31)_ = 13.089, *p* = .001, η^2^_p_ = .297. Additionally, there was a significant expectancy × target interaction, *F*_(1.43)_ = 81.530, *p* < .001, η^2^_p_ = .725, and a significant time × expectancy × target interaction, *F*_(5.144)_ = 3.752, *p* = .004, η^2^_p_ = .108. Post-hoc pairwise comparisons showed that when participants expected to gain, they reacted significantly faster to loss targets in blocks 3 and 4 than in block 1 (block 1 vs. block 3: *p* = .001, block 1 vs. block 4: *p* < .001) and faster in block 4 than in block 2 (*p* = .032). When participants expected to lose, they reacted significantly faster to gain targets in blocks 2, 3, and 4 than block 1 (block 1 vs. block 2: *p* < .001, block 1 vs. block 3: *p* < .001, block 1 vs. block 4: *p* < .001) and faster in block 4 than block 2 (*p* = .022).

**Conclusion**

In line with our optimism robustness hypothesis, analyses of the evolution of RTs in incongruent conditions showed that when participants received disconfirming rewarding feedback while being pessimistic, they updated their attention quite rapidly (from the first to the second block, a steep learning curve). In contrast, when participants received disconfirming punishing feedback while being optimistic, they updated their attention more slowly over the course of the experiment (only in the third and fourth blocks, a flat learning curve). These results underscore the robustness of optimistic expectancies compared with pessimistic expectancies when being confronted with disconfirming feedback.
