# Supplementary figures and images for: Seeing through rose-colored glasses: How optimistic expectancies guide visual attention
Source: PLoS One. 2018 Feb 21;13(2):e0193311. doi: 10.1371/journal.pone.0193311 (PMC5821386; doi:10.1371/journal.pone.0193311)

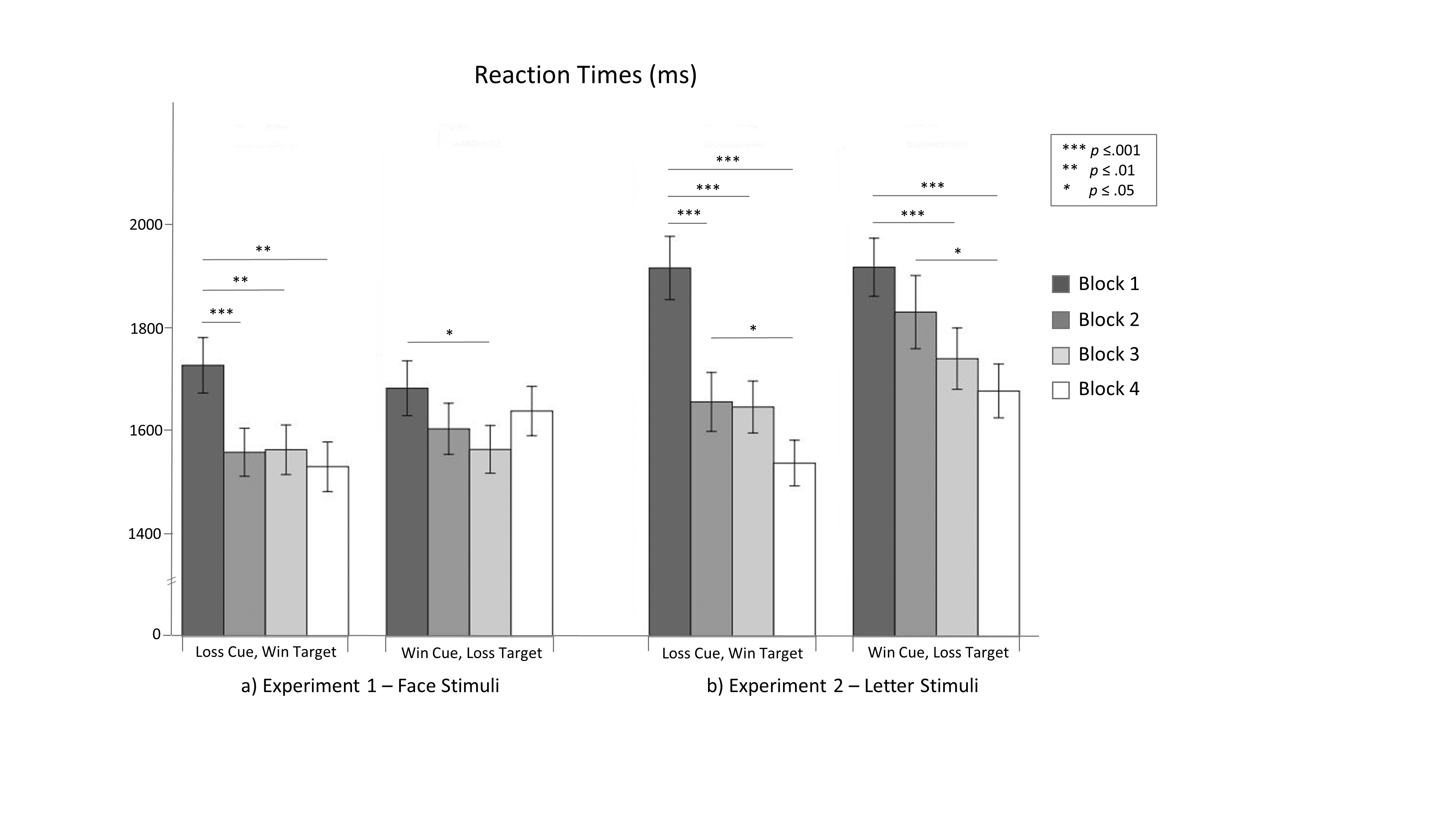

Supplement: S1 Fig — To simplify the graph, the remaining conditions are not depicted although statistics were run on all experimental conditions. The error bars depict standard errors. (TIF) [file pone.0193311.s003.tif]
