# Supplementary material for: Seeing through rose-colored glasses: How optimistic expectancies guide visual attention
Source: PLoS One. 2018 Feb 21;13(2):e0193311. doi: 10.1371/journal.pone.0193311 (PMC5821386; doi:10.1371/journal.pone.0193311)
Supplement: S1 Table — (DOCX) [file pone.0193311.s004.docx]

**S1 Table.** **Mean values, standard errors, and 95 % confidence intervals (CIs) of pupil diameter change for the three expectancy cue conditions during five analyzed 0.5-s time intervals following cue onset in Experiments 1 (N = 31) and 2 (N = 32).**

| **Condition** |  | **Exp. 1** | | | | | **Exp. 2** | | | | |
| --- | --- | --- | --- | --- | --- | --- | --- | --- | --- | --- | --- |
|  |  | 0.0  - 0.5 | 0.5  - 1.0 | 1.0  - 1.5 | 1.5  - 2.0 | 2.0  - 2.5 | 0.0  - 0.5 | 0.5  - 1.0 | 1.0  - 1.5 | 1.5  - 2.0 | 2.0  - 2.5 |
| Gain Cue | M  SE | - .004  .003 | - .025  .006 | .003  .009 | .003  .009 | - .005  .008 | - .001  .004 | - .004  .007 | .029  .012 | .024  .015 | .014  .012 |
|  | 95 %  CI | - .011,  .003 | - .037,  - .013 | - .015,  .022 | - .015,  .021 | - .021,  .011 | - .008,  .006 | - .018,  .010 | .004,  .053 | - .006,  .054 | - .010,  .038 |
| Loss Cue | M  SE | .000  .003 | - .021  .006 | - .003  .009 | - .005  .010 | - .013  .010 | - .005  .002 | - .005  .007 | .027  .012 | .034  .013 | .021  .011 |
|  | 95 %  CI | - .007,  .007 | - .033,  - .010 | - .021,  .015 | - .025,  .015 | - .033,  .006 | - .010,  - .001 | - .018,  .009 | .001,  .052 | .008,  .061 | - .001,  .043 |
| Ambiguous Cue | M  SE | - .005  .004 | - .052  .008 | - .042  .014 | - .025  .013 | - .034  .013 | - .006  .004 | - .027  .008 | - .004  .014 | .007  .016 | - .019  .012 |
|  | 95 %  CI | - .013,  .004 | - .069,  - .036 | - .070,  - .014 | - .052,  .002 | - .062,  - .007 | - .015,  .003 | - .044,  - .009 | - .032,  .024 | - .027,  .040 | - .043,  .006 |
