# Supplementary material for: Seeing through rose-colored glasses: How optimistic expectancies guide visual attention
Source: PLoS One. 2018 Feb 21;13(2):e0193311. doi: 10.1371/journal.pone.0193311 (PMC5821386; doi:10.1371/journal.pone.0193311)
Supplement: S2 Table — (DOCX) [file pone.0193311.s005.docx]

**S2 Table.** **Mean values, standard errors, and 95 % confidence intervals (CIs) of reaction times, time to first hit, and percentage of gazing at the target half a second after the first hit are summarized for all experimental conditions in Experiments 1 and 2.**

| **Condition** |  | **Reaction times (in ms)** | | **Time to first hit**  **(in ms)** | | **Percentages of gazing at the target half a second after the first hit (in %)** | |
| --- | --- | --- | --- | --- | --- | --- | --- |
|  |  | *Exp. 1 (N = 31)* | *Exp. 2 (N = 32)* | *Exp. 1*  *(N = 31)* | *Exp. 2 (N = 32)* | *Exp. 1*  *(N = 31)* | *Exp. 2*  *(N = 32)* |
| Gain cue | M  SE | 1585.47  35.51 | 1476.27  35.60 | 1137.37  15.64 | 1082.26  25.28 | 77.10  1.20 | 66.10  1.70 |
|  | 95 % CI | 1512.94,  1657.99 | 1403.67,  1548.87 | 1105.44,  1169.31 | 1030.70,  1133.82 | 74.70,  79.50 | 62.60,  69.60 |
| Loss cue | M  SE | 1568.11  34.79 | 1459.27  32.10 | 1118.89  18.24 | 1073.00  22.44 | 76.30  1.20 | 67.70  1.90 |
|  | 95 % CI | 1497.06,  1639.16 | 1393.81,  1524.73 | 1081.63,  1156.14 | 1027.22,  1118.76 | 73.80,  78.80 | 63.90,  71.40 |
| Ambiguous cue | M  SE | 1566.54  33.92 | 1595.50  40.59 | 1137.16  18.10 | 1128.03  24.50 | 77.90  1.40 | 65.70  1.80 |
|  | 95 % CI | 1497.26,  1635.82 | 1512.72,  1678.29 | 1100.20,  1174.13 | 1078.07,  1177.99 | 75.00,  80.80 | 61.90,  69.40 |
| Gain target | M  SE | 1560.80  34.82 | 1451.49  38.72 | 1129.71  18.53 | 1058.89  25.29 | 77.80  1.30 | 68.30  1.70 |
|  | 95 % CI | 1489.68,  1631.92 | 1372.52,  1530.46 | 1091.88,  1167.55 | 1007.31,  1110.46 | 75.10,  80.50 | 64.80,  71.80 |
| Loss target | M  SE | 1585.95  34.80 | 1569.21  37.70 | 1132.57  15.84 | 1129.97  25.21 | 76.40  1.20 | 64.70  1.90 |
|  | 95 % CI | 1514.88,  1657.02 | 1492.32,  1646.09 | 1100.22,  1164.92 | 1078.55,  1181.39 | 74.00,  78.90 | 60.80,  68.50 |
| Gain cue,  gain target | M  SE | 1546.18  33.88 | 1171.08  39.62 | 1131.92  18.88 | 875.00  28.38 | 79.40  1.20 | 73.30  1.90 |
|  | 95 % CI | 1476.98,  1615.38 | 1090.28,  1251.87 | 1093.37,  1170.47 | 817.11,  932.89 | 77.00,  81.80 | 69.40,  77.30 |
| Gain cue,  loss target | M  SE | 1624.75  39.90 | 1781.46  52.90 | 1142.82  16.86 | 1289.52  36.32 | 74.80  1.50 | 58.90  2.20 |
|  | 95 % CI | 1543.26,  1706.23 | 1673.58,  1889.34 | 1108.39,  1177.26 | 1215.45,  1363.59 | 71.80,  77.80 | 54.50,  63.30 |
| Loss cue,  gain target | M  SE | 1585.62  39.87 | 1678.16  45.67 | 1118.60  21.87 | 1227.47  32.37 | 75.30  1.60 | 64.00  2.00 |
|  | 95 % CI | 1504.20,  1667.04 | 1585.01,  1771.31 | 1073.93,  1163.28 | 1161.46,  1293.49 | 72.00,  78.70 | 59.90,  68.20 |
| Loss cue,  loss target | M  SE | 1550.61  32.17 | 1240.38  43.03 | 1119.17  18.03 | 918.50  32.24 | 77.20  1.10 | 71.30  2.20 |
|  | 95 % CI | 1484.91,  1616.30 | 1152.62,  1328.14 | 1082.34,  1156.00 | 852.75,  984.25 | 74.90,  79.60 | 66.80,  75.80 |
| Ambiguous cue, gain target | M  SE | 1550.59  35.26 | 1505.23  53.17 | 1138.61  21.70 | 1074.18  34.05 | 78.60  1.80 | 67.60  1.90 |
|  | 95 % CI | 1478.59,  1622.59 | 1396.78,  1613.67 | 1094.31,  1182.92 | 1002.70,  1145.67 | 75.00,  82.20 | 63.30,  71.60 |
| Ambiguous cue, loss target | M  SE | 1582.49  36.29 | 1685.78  43.50 | 1135.71  20.91 | 1181.88  30.20 | 77.20  1.40 | 63.80  2.20 |
|  | 95 % CI | 1508.37,  1656.61 | 1597.06,  1774.50 | 1093.01,  1178.42 | 1120.29,  1243.47 | 74.40,  80.00 | 59.20,  68.30 |
