# Supplementary material for: Seeing through rose-colored glasses: How optimistic expectancies guide visual attention
Source: PLoS One. 2018 Feb 21;13(2):e0193311. doi: 10.1371/journal.pone.0193311 (PMC5821386; doi:10.1371/journal.pone.0193311)
Supplement: S3 Table — (DOCX) [file pone.0193311.s006.docx]

**S2 Table. Difference scores of reaction times, time to first hit, and percentage of gazing at the target half a second after the first hit are summarized for Experiments 1 and 2.**

| **Difference score** | **Reaction times (in ms)** | | **Time to first hit (in ms)** | | **Percentage of gazing at the target half a second after the first hit (in %)** | |
| --- | --- | --- | --- | --- | --- | --- |
|  | Exp. 1 | Exp. 2 | Exp. 1 | Exp. 2 | Exp. 1 | Exp. 2 |
| [Gain cue, loss target] –[Gain cue, gain target] | 78.57 | 610.39 | 10.90 | 414.52 | -4.60 | -14.46 |
| [Loss cue, gain target] –[Loss cue, loss target] | 35.02 | 437.78 | -.56 | 308.97 | -1.90 | -7.29 |
