# Supplementary material for: Seeing through rose-colored glasses: How optimistic expectancies guide visual attention
Source: PLoS One. 2018 Feb 21;13(2):e0193311. doi: 10.1371/journal.pone.0193311 (PMC5821386; doi:10.1371/journal.pone.0193311)
Supplement: S4 Table — Significant p-values are marked with an asterisk. (DOCX) [file pone.0193311.s007.docx]

**S4 Table.** **Statistical values from the 3 (expectancy: gain, loss, ambiguous) x 5 (time: 0-0.5 s, 0.5-1 s, 1-1.5 s, 1.5-2 s, 2-2.5 s) ANOVA are given for the pupil diameter change analysis from Experiments 1 and 2.**

| **3x5 ANOVA** | **Pupil diameter change** | |
| --- | --- | --- |
|  | *Exp. 1* | *Exp. 2* |
| Main effect: expectancy | *F*_1,44_ = 11.854  *p* ≤ .001*  η^2^_p_ = .283 | *F*_2,56_ = 12.438  *p* ≤ .001*  η^2^_p_ = .308 |
| Main effect: time | *F*_2,50_ = 4.098  *p* = .029*  η^2^_p_ = .120 | *F*_2,43_= 4.284  *p* = .029*  η^2^_p_ = .133 |
| Expectancy  × time interaction | *F*_5,148_ = 9.052  *p* ≤ .001*  η^2^_p_ = .232 | *F*_4,120_ = 4.988  *p* = .001*  η^2^_p_ = .151 |

Significant *p*-values are marked with an asterisk.
