# Supplementary material for: Seeing through rose-colored glasses: How optimistic expectancies guide visual attention
Source: PLoS One. 2018 Feb 21;13(2):e0193311. doi: 10.1371/journal.pone.0193311 (PMC5821386; doi:10.1371/journal.pone.0193311)
Supplement: S5 Table — Significant p-values are marked with an asterisk. (DOCX) [file pone.0193311.s008.docx]

**S5 Table.** **Statistical values from the 3 (expectancy: gain, loss, ambiguous) x 2 (target: gain, loss) ANOVA are given for the reaction time analysis, time to first hit analysis, and percentage of gazing at the target half a second after the first hit analysis from Experiments 1 and 2.**

| **3x2 ANOVA** | **Reaction time analysis** | | **Time to first hit analysis** | | **Percentage of gazing at the target half a second after the first hit analysis** | |
| --- | --- | --- | --- | --- | --- | --- |
|  | *Exp. 1* | *Exp. 2* | *Exp. 1* | *Exp. 2* | *Exp. 1* | *Exp. 2* |
| Main effect: expectancy | *F*_2,60_ = 1.686  *p* = .194  η^2^_p_ = .053 | *F*_1,46_ = 28.227  *p* ≤ .001*  η^2^_p_ = .477 | *F*_2,60_ = 1.767  *p* = .180  η^2^_p_= .056 | *F*_2,62_ = 4.918  *p* = .010*  η^2^_p_= .137 | *F*_2,60_ = 1.665  *p* = .198  η^2^_p_= .053 | *F*_2,62_ = 1.533  *p* = .224  η^2^_p_= .047 |
| Main effect: target | *F*_1,30_ = 3.292  *p* = .080  η^2^_p_= .099 | *F*_1,31_ = 12.582  *p* = .001*  η^2^_p_= .289 | *F*_1,30_ = .054  *p* = .818  η^2^_p_= .002 | *F*_1,31_ = 7.247  *p* = .011*  η^2^_p_= .189 | *F*_1,30_ = 2.123  *p* = .155  η^2^_p_= .066 | *F*_1,31_ = 7.464  *p* = .010*  η^2^_p_= .194 |
| Expectancy  × target interaction | *F*_2,60_ = 8.324  *p* = .001*  η^2^_p_= .217 | *F*_1,43_ = 79.723  *p* ≤ .001*  η^2^_p_= .720 | *F*_2,60_ = .164  *p* = .849  η^2^_p_= .005 | *F_2_*_,49_ = 72.432  *p* ≤ .001*  η^2^_p_= .700 | *F*_2,50_ = 7.482  *p* = .002*  η^2^_p_= .200 | *F*_2,50_ = 31.007  *p* < .001*  η^2^_p_= .500 |

Significant *p*-values are marked with an asterisk.
