# Supplementary material for: Seeing through rose-colored glasses: How optimistic expectancies guide visual attention
Source: PLoS One. 2018 Feb 21;13(2):e0193311. doi: 10.1371/journal.pone.0193311 (PMC5821386; doi:10.1371/journal.pone.0193311)
Supplement: S6 Table — Significant p-values are marked with an asterisk. (DOCX) [file pone.0193311.s009.docx]

**S6 Table.** ***P*-values from post-hoc pairwise *t*-tests (Sidak corrected) comparing pupil diameter change for the different expectancy cues during five 0.5-s time intervals following cue onset in Experiments 1 and 2.**

| **Post-hoc t-tests** | | | **Exp. 1** | | | | | **Exp. 2** | | | | |
| --- | --- | --- | --- | --- | --- | --- | --- | --- | --- | --- | --- | --- |
|  |  |  | 0.0  -0.5 | 0.5  -1.0 | 1.0  -1.5 | 1.5  -2.0 | 2.0  -2.5 | 0.0  -0.5 | 0.5  -1.0 | 1.0  -1.5 | 1.5  -2.0 | 2.0  -2.5 |
| Main effect expec-tancy | Gain cue | Loss cue | .352 | .898 | .410 | .314 | .437 | .676 | 1.000 | .990 | .381 | .720 |
|  | Gain cue | Amb. cue | 1.000 | .005* | < .001* | .002* | .004* | .713 | .080 | < .001* | .009* | < .001* |
|  | Loss cue | Amb. cue | .710 | .001* | .001* | .057 | .072 | .999 | .005* | .005* | .005* | < .001* |

Significant *p*-values are marked with an asterisk.
