# Supplementary material for: Seeing through rose-colored glasses: How optimistic expectancies guide visual attention
Source: PLoS One. 2018 Feb 21;13(2):e0193311. doi: 10.1371/journal.pone.0193311 (PMC5821386; doi:10.1371/journal.pone.0193311)
Supplement: S7 Table — Significant p-values are marked with an asterisk. (DOCX) [file pone.0193311.s010.docx]

**S7 Table.** ***P*-values from post-hoc pairwise *t*-tests (Sidak corrected) comparing different experimental conditions are given for reaction time analysis, time to first hit analysis, and percentage of gazing at the target half a second after the first hit analysis from Experiment 1 and 2.**

| **Post-hoc t-tests** | | | **Reaction time analysis** | | **Time to first hit analysis** | | **Percentage of gazing at the target half a second after the first hit analysis** | |
| --- | --- | --- | --- | --- | --- | --- | --- | --- |
|  |  |  | *Exp. 1* | *Exp. 2* | *Exp. 1* | *Exp. 2* | *Exp. 1* | *Exp. 2* |
| Main effect expectancy | Gain cue | Loss cue | .446 | .500 | .245 | .905 | .693 | .435 |
|  | Gain cue | Amb. cue | .364 | < .001* | 1.000 | .085 | .780 | .983 |
|  | Loss cue | Amb. cue | .997 | < .001* | .406 | .037* | .233 | .267 |
| Expectancy × target interaction | Gain target | Gain vs. loss cue | .191 | < .001* | .784 | < .001* | .009* | < .001* |
|  |  | Gain vs. amb. cue | .993 | < .001* | .969 | < .001* | .899 | .001* |
|  |  | Loss vs. amb. cue | .117 | < .001* | .606 | < .001* | .117 | .104 |
|  | Loss target | Gain vs. loss cue | .001* | < .001* | .355 | < .001* | .163 | < .001* |
|  |  | Gain vs. amb. cue | .143 | .015* | .979 | .003* | .066 | .096 |
|  |  | Loss vs. amb. cue | .041* | < .001* | .657 | < .001* | 1.000 | < .001* |
|  | Gain vs. loss target | Gain cue | .001* | < .001* | .536 | < .001* | .001* | < .001* |
|  |  | Loss cue | .093 | < .001* | .973 | < .001* | .180 | .001* |
|  |  | Amb. cue | .171 | .002* | .898 | .019* | .339 | .063 |

Significant *p*-values are marked with an asterisk.
