# Supplementary material for: Seeing through rose-colored glasses: How optimistic expectancies guide visual attention
Source: PLoS One. 2018 Feb 21;13(2):e0193311. doi: 10.1371/journal.pone.0193311 (PMC5821386; doi:10.1371/journal.pone.0193311)
Supplement: S8 Table — (DOCX) [file pone.0193311.s011.docx]

**S8 Table. Number (percentage) of participants answering “yes” and “no” to questions on the post-experimental questionnaire in Experiments 1 and 2.**

| **Question** | **Experiment 1** | | **Experiment 2** | |
| --- | --- | --- | --- | --- |
|  | yes | no | yes | no |
| Did you pay attention to probability cues in the beginning of each trial? | 23 (74 %) | 8  (26 %) | 28 (88 %) | 4 (12 %) |
| Were probability cues in the beginning of each trial important to prepare your answer? | 9  (29 %) | 22  (71 %) | 27 (84 %) | 5 (16 %) |
| Do you feel that probability cues helped you to answer as fast and correct as possible? | 9  (29 %) | 22 (71 %) | 25 (78 %) | 7 (22 %) |
| Did probability cues influence difficulty to find the gain or loss targets? | 11 (36 %) | 20 (64 %) | 24 (75 %) | 8 (25 %) |
